# High surface area and interconnected nanoporosity of clay-rich astromaterials

**DOI:** 10.1038/s41598-024-61114-2

**Published:** 2024-05-06

**Authors:** Laurence A. J. Garvie, László Trif, Desireé Cotto-Figueroa, Erik Asphaug, Christian G. Hoover

**Affiliations:** 1 Buseck Center for Meteorite Studies, Arizona State University, 781 East Terrace Rd., Tempe, AZ 85287‑6004 USA; 2 School of Earth and Space Exploration, Arizona State University, 781 East Terrace Rd., Tempe, AZ 85287‑6004 USA; 3 Institute of Materials and Environmental Chemistry, Research Center for Natural Sciences, Magyar tudósok körútja 2, Budapest, 1117 Hungary; 4 Department of Physics and Electronics, The University of Puerto Rico at Humacao, Call Box 860, Humacao, PR 00792 USA; 5 Lunar and Planetary Laboratory, University of Arizona, PO Box 210092, Tucson, AZ 85721 USA; 6 School of Sustainable Engineering and the Built Environment, Ira A. Fulton Schools of Engineering, Arizona State University, Tempe, AZ 85287 USA

**Keywords:** Planetary science, Astronomy and planetary science, Materials science

## Abstract

Porosity affects key astromaterial processes from disruption in our atmosphere and impact with the ground, to the comminution of boulders by thermal and impact processes and slope mechanics on asteroid surfaces, to access and utilization of in-situ resources. Whereas the bulk porosity of clay-rich meteorites is well established, the magnitude of their surface area and nano-scale porosity is poorly known. Here we use N_2_ BET gas adsorption to measure the specific surface area and nanoscale pore distribution in several clay-rich meteorites. Two recent falls Tarda (C2-ung) and Aguas Zarcas (CM2) have specific surface areas of 72.5 and 16.5 m^2^/g, respectively. However, the specific surface area of Tarda ranges from 33.7 to 81.6 m^2^/g depending on outgassing conditions. The Tarda surface area is dominated by an interconnected network of ~ 3-nm-sized pores, whereas Aguas Zarcas shows a lower density of ~ 3 nm pores and broader size distribution around 40 nm. In contrast, Ivuna and Orgueil (CI1) have surface areas of ~ 15 to 18 m^2^/g: the low values compared to Tarda are likely due to the neoformation of pore-blocking minerals during atmospheric exposure. These data suggest that returned samples from asteroids Ryugu and Bennu, which are mineralogically and texturally similar to Tarda, also have interconnected nano-scale porosity with high surface area.

## Introduction

Clay-rich rocks have high surface areas and complex pore structures controlled by the microstructure and aggregation of the clays. Pore sizes and shapes vary across a range of dimensions and scales from nanometer-sized to larger micron-sized pores between clay aggregates^1,2^. Although there is significant research on the bulk porosity of meteorites^3–6^, the surface area and bulk submicron-scale porosity in the carbonaceous chondrite (CC) meteorites are relatively understudied. This porosity, pore-size distribution, and pore connectivity are important properties in clay-rich rocks as they affect astromaterial properties including their strength, thermal conductivity, speed and attenuation of sound^5^, and transport of fluids on the early parent body that gives rise to their complex, multiscale mineralogy, e.g.^7,8^. The bulk fine-scale porosity and pore-size distribution can be probed through a range of techniques including X-ray tomography, transmission electron microscopy (TEM), nuclear magnetic resonance (NMR) cryoporometry, and inert gas adsorption^9–13^. Of these methods, physical adsorption of an inert gas, such as nitrogen is widely used to probe the surface area, bulk fine-scale porosity, and pore-size distribution^13^.

Nitrogen gas adsorption provides insights into the surface area and pore characteristics of pores with a diameter less than ~ 200 nm. Insights into the adsorption process are provided by the application of the Brunauer–Emmett–Teller (BET) theory^13–16^. Pore sizes accessible to gas adsorption are divided into micropores (< 2 nm), mesopores (2–50 nm), and macropores (50 to 200 nm)^16^. The BET and the Barrett, Joyner, and Halenda (BJH) methods are used to create pore volume and surface area distributions based on adsorption–desorption isotherms in a wide range of terrestrial clays and shales^1,17–20^. However, only limited BET gas adsorption research has been carried out on meteorites rich in clay^21–23^. Consequently, the surface area and nanopore-size distribution in these meteorites, and by extension their parent asteroids, remain poorly understood.

Many petrologic types 1 and 2 CCs, including the samples returned from asteroids Ryugu and Bennu, are dominated by phyllosilicates^24–29^. Bulk powder low-angle X-ray diffraction (XRD) shows that their clays are broadly divided into serpentines and smectites or a combination thereof^25,30–32^. Before entering the Earth’s atmosphere, meteorites and asteroid-returned samples inhabit the ultrahigh vacuum of space, with pressure near 1.3 × 10^–11^ Pa, equivalent to at most a few tens of atoms/cm^3^. However, once on Earth, their pristine surface area is exposed to atmospheric gases. As a result, clay-rich meteorites can undergo rapid terrestrial alteration given their high porosity and the ease with which the submicron-scale minerals interact with our atmosphere. Therefore, it can be challenging to determine, for example, their indigenous surface area and porosity given the ease with which this is altered by atmospheric exposure, especially for meteorites that have had significant residence time on Earth.

In this study, we measure the specific surface area, fine-scale porosity, and pore-size distribution in clay-rich CC meteorites with N_2_ BET gas adsorption and apply the BJH analysis to create pore volume and surface area distributions based on the adsorption–desorption isotherms. We address the challenges associated with making these measurements given the rapidity with which clay-rich meteorites absorb atmospheric water by measuring the N_2_ BET isotherms for samples subjected to a range of outgassing conditions. Further, the identity and quantity of atmospherically adsorbed gases are measured by thermal gravimetric (TG) analysis combined with mass spectrometric evolved-gas analysis (MSEGA).

Our primary meteorite of study is the Tarda (C2-ung) fall. This is the most recent smectite-rich meteorite to fall and be collected here on Earth. It was rapidly collected and curated under a dry N_2_ atmosphere. As such, it is one of the least terrestrially contaminated clay-rich meteorites in our collections and is thus ideally suited to study both its indigenous physical properties as well as the effects of atmospheric exposure. For comparison, BET data is also acquired from four well-studied clay-rich CC falls, Ivuna (CI1), Orgueil (CI1), Aguas Zarcas (CM2), and Murchison (CM2), and an anhydrous CC, Allende (CV3).

A major finding of our study is that Tarda has a specific surface area of 72.5 m^2^/g after outgassing at 110 °C for 24 h under vacuum, and pore-size distribution, obtained by applying the BJH method from the adsorption isotherm, with a maximum near the upper boundary with the micropore range and a broad tail into the macropore region. In contrast, the other clay-rich meteorites have specific surface areas of 13.8 to 18.6 m^2^/g, and bimodal pore-size distributions with less-pronounced maximum near 3 nm and broader more intense distribution that straddles the meso- macropore boundary. The Tarda data advocates for a high density of pores, in, around, and between the nano-sized aggregates of poorly ordered clays. It is speculated that the recently returned asteroid samples from Ryugu and Bennu, which show mineralogical similarities to Tarda, will also show a similarly high surface area and fine-scale, interconnected nanoporosity.

## Results and discussion

### Nitrogen adsorption and surface area measurements

Before conducting the N_2_ BET measurements, it is necessary to remove physisorbed species through outgassing^13^. In this paper, the outgassing pretreatment is listed after the meteorite name: Tarda-250 refers to Tarda outgassed under flowing dry N_2_ at 250 °C for 24 h; Tarda-110 V, outgassed at 110 °C under high vacuum for 24 h; Tarda-100, outgassed at 100 °C under flowing N_2_ for 24 h; and Tarda-VRT, outgassed under vacuum at room temperature for 24 h. Tarda-NH is the sample measured without outgassing and after prolonged curation under a dry N_2_ atmosphere. The BET measurements are made by exposing the sample to N_2_ at a series of controlled pressures while maintaining the sample at -195.8 °C, which corresponds to the boiling point of N_2_. The volume of adsorbed gas is measured over a relative equilibrium adsorption pressure (p/p^0^) from near 0 to 1, where p is the absolute equilibrium pressure and p^0^ is the saturation pressure of the gas^1,13^. The plot of p/p^0^ versus the quantity of gas adsorbed is called the adsorption isotherm. Before the N_2_ BET analyses, the samples were characterized by powder X-ray diffraction (See Supplementary Data, Figs. S1, S2, and details in the Experimental section).

**Figure 1:**
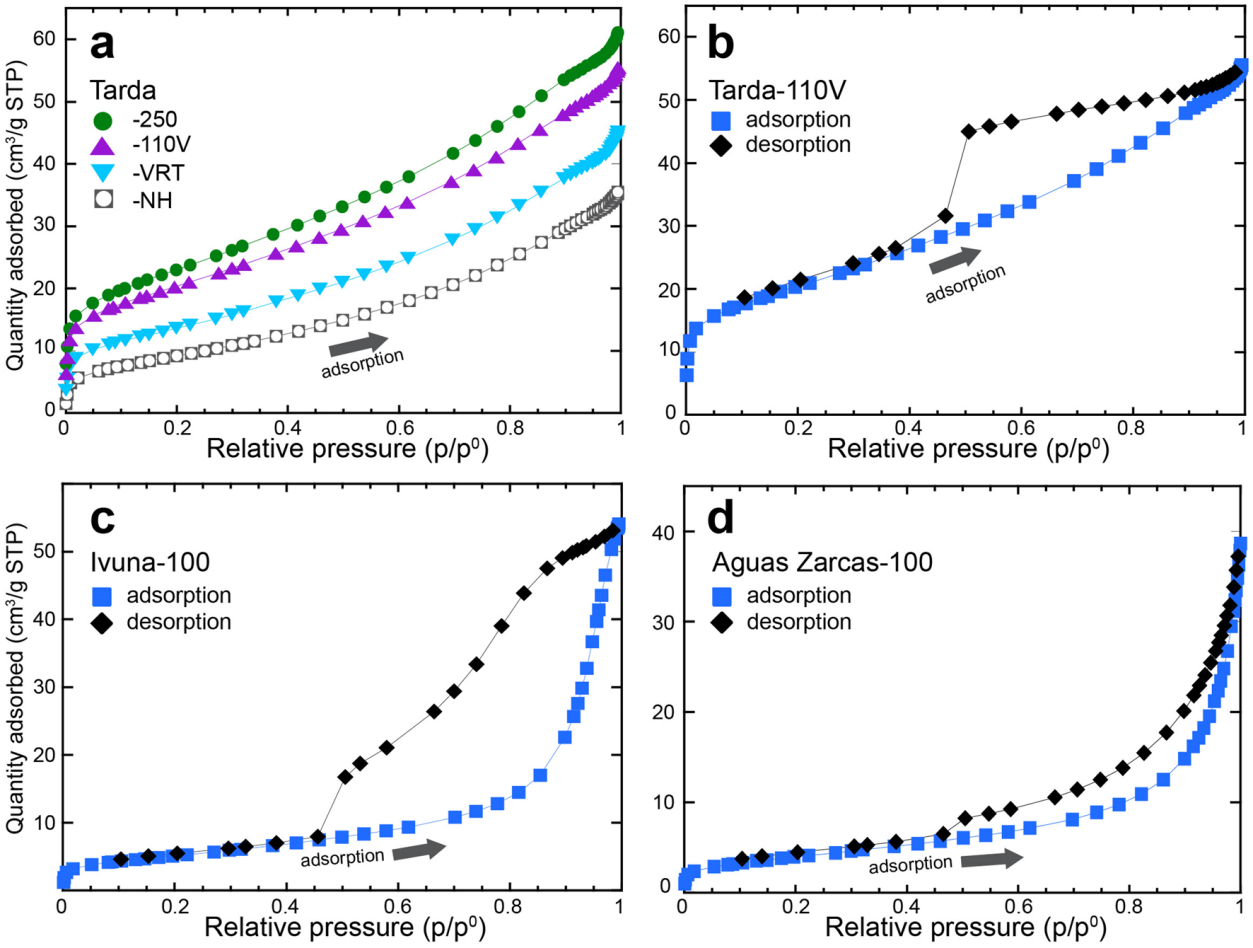
Isotherms measured at 77 K for selected meteorites. (**a**) Adsorption isotherms for Tarda after different outgassing pretreatments. (**b**) Adsorption–desorption isotherms for Tarda-110 V. (**c**) Ivuna-100, and (**d**) Aguas Zarcas-100. Outgassing conditions are: -250, heating to 250 °C under flowing N_2_ for 24 h; -110 V, heating to 110 °C under vacuum for 24 h; -100, heating to 100 °C under flowing N_2_ for 24 h; -VRT, kept under vacuum at room temperature for 24 h; and -NH, sample not outgassed and run directly from the N_2_ storage.

The Tarda adsorption isotherms show six distinct regions (Fig. 1a) – a near vertical rise in adsorption for p/p^0^ < 0.02 (Fig. S3), a broad kink in the adsorption volume up to p/p^0^ ~ 0.05, an almost linear increase in p/p^0^ to ~ 0.35, slightly non-linear positive adsorption to p/p^0^ ~ 0.9, after which there is a slight flattening for p/p^0^ between 0.9 and 0.95, followed by an asymptotic increase in adsorption at p/p^0^ = 1 (Fig. 1a). Comparison of these isotherms with the Brunauer-Deming-Deming-Teller (BDDT) physisorption isotherm types^13,16^ shows that Tarda possesses a hybrid adsorption isotherm with a Type 1b onset followed by a Type II isotherm with a hint of Type IV character at high p/p^0^. A Type Ib onset is interpreted as the filling of micropores with a range of pore volumes that extends into the mesopore region. The steep, almost linear increase in adsorption above p/p^0^ ~ 0.05, suggests a broad range of mesopore volumes. The final asymptotic increase at p/p^0^ = 1 can be attributed to the filling of macropores, i.e., those > 200 nm in diameter as p/p^0^ approaches 1. In general, the degree of N_2_ uptake for p/p^0^ ~ 1 is proportional to the total porosity of pores to ~ 200 nm in diameter showing that outgassing significantly increases the total micro and mesoporosity. While the Tarda adsorption isotherms do not have a plateau at high p/p^0^, as is expected for a Type IV isotherm, the slight flattening for p/p^0^ between 0.9 and 0.95 suggests complete filling of the mesopores up to ~ 200 nm, but a lower density of large macropores remain unfilled at p/p^0^ = 1.

Tarda shows pronounced hysteresis during desorption (Fig. 1b, S4a), dominated by an H2 hysteresis loop pattern, though the onset of the desorption for p/p^0^ > 0.95 shows an H3 character^13^. The desorption isotherm does not track with the adsorption path and shows a marked hysteresis at p/p^0^ ~ 0.35–0.45. The steepness of the desorption branch for p/p^0^ < 0.5 informs on the pore-size range, pore geometry, and connectivity (see below).

The maximum quantity of gas adsorbed increases from Tarda NH → VRT → 100/110 V → 250 indicating progressive desorption of adsorbed gases with increasing temperature, and hence a larger surface area for the adsorption of N_2_ during the BET experiments. Outgassing is typically done by heating the sample under a vacuum^13^: the temperature is chosen depending on the nature of the sample. Here, the adsorption–desorption curves for the sample outgassed at 100 °C under flowing dry nitrogen (Tarda-100) and that outgassed at 110 °C for 24 h under vacuum (Tarda-110 V) overlap (Fig. S4b). Therefore, for the clay-rich meteorites studied here the outgassing under flowing dry N_2_ at 100 °C removes a similar quantity of physisorbed species as heating under a vacuum at 110 °C.

The adsorption profiles for the smectite-rich Orgueil and Ivuna and serpentine-rich Aguas Zarcas and Murchison outgassed at 100 °C under flowing N_2_, are similar (Figs. 1c, d, S5, S6) and match a Type II isotherm. There is a small but rapid increase in adsorbed N_2_ for p/p^0^ < 0.02, gradual uptake up to p/p^0^ ~ 0.8, and then rapid uptake to p/p^0^ = 1. Aguas Zarcas and Murchison show an H3-type desorption isotherm with a rapid decrease in the desorption isotherm at 0.4 < p/p^0^ < 0.5 (Fig. 1d, S6). The hysteresis loop of the desorption branch for Ivuna and Orgueil is more complex. Desorption of Orgueil is linear for 0.5 < p/p^0^ < 1 with a sharp and sudden decrease from p/p^0^ of 0.5 to 0.45 (Fig. S5). Ivuna shows linear desorption 0.85 < p/p^0^ < 1, H2b-like hysteresis in the range 0.5 < p/p^0^ < 0.85, and rapid decrease in the quantity adsorbed and closure of desorption isotherm at 0.4 < p/p^0^ < 0.5 (Fig. 1c). In contrast, Allende, which is dominated by anhydrous silicates, Fe sulfides, and metal shows a Type II adsorption isotherm and a desorption branch that largely tracks the adsorption pathway (Fig. S7).

Insights into the surface area of porous materials can be gained by application of the Brunauer–Emmett–Teller (BET) equation to the isotherms

$$\frac{1}{{{\varvec{n}}\left( {\frac{{p^{0} }}{p} - 1} \right)}} = \frac{C - 1}{{{\varvec{n}}_{m} C}}\left( {\frac{p}{{p^{0} }}} \right) + \frac{1}{{{\varvec{n}}_{m} C}}$$

where **n** is the specific amount adsorbed in cm^3^/g at STP at relative pressure p/p^0^ and **n**_m_ is the specific monolayer capacity^13,16^. The specific monolayer capacity **n**_m_ is determined by plotting the BET function 1/(**n**(p^0^/p-1) against the relative adsorption pressure p/p^0^, called the BET plot^13^, within the linear range of p/p^0^, which is typically ∼0.05–0.30 for Type II and Type IVa isotherms^13^. The slope of the linear regression of the linear range of the BET plot is used to calculate **n**_m_ = 1/(*s* + *i*), where *s* is the slope *i* is the intercept and C = *s*/*i* + 1. The suitability of this linear BET plot is demonstrated for Tarda-100, which shows that p/p^0^ from 0.30074 to 0.056479 lie along a straight line with R^2^ = 0.99993 (Fig. S8 and is the region of the isotherm in which statistically the volume adsorbed corresponds to just the complete monolayer. Above and below these p/p^0^ values the points deviate from the linear regression (Fig. S8) and are not used for the BET surface area calculation. The linear range for each plot is available in the online datasets^33^. The BET specific surface area is calculated using,

$${S}_{BET} =\frac{{{\varvec{n}}}_{m}\mathrm{ L }{\sigma }_{m}}{{V}_{0} m}$$

where *S*_BET_ is the BET-specific area, **n**_m_ the monolayer capacity, σ_m_ is the molecular cross-sectional area occupied by the adsorbate molecule, which for N_2_ is 16.2 × 10^–20^ m^2^ (0.162 nm^2^), L is the Avogadro constant 6.022 × 10^23^, V_0_ is the molar gas volume of the adsorptive at STP, and m is the mass of the sample. Using this equation, the S_BET_ of the clay-rich meteorites range from 10.27 to 81.61 m^2^/g but only 1.21 m^2^/g for the anhydrous Allende (Table 1). Different outgassing treatments have significant effects on S_BET_ with the Tarda values ranging from 33.67 m^2^/g for the sample taken directly from the nitrogen cabinet to 81.61 m^2^/g after the 250 °C treatment. Despite the differing hysteresis patterns between the smectite-rich Orgueil and Ivuna and the serpentine-rich Aguas Zarcas and Murchison, their *S*_BET_ values are close (Table 1), though the profile differences provide information on pore-size distribution, pore geometry, and network effects.

**Table 1:** Meteorites studied, outgas conditions, BET surface area (S_BET_), BET C parameter (C), monolayer capacity—**n**_m_, BJH adsorption cumulative volume of pores (V_ad_), BJH desorption cumulative volume of pores (V_ds_), and pore size for selected meteorites determined from the N_2_ adsorption isotherms at 77 K.

Meteorite	Outgas condition	C	n_m_ (cm^3^/g)	S_BET_ (m^2^/g)	V_ad_ (cm^3^/g)^1^	V_ds_ (cm^3^/g)^2^	Pore size^3^ nm
Tarda	250	189.3	18.7499	81.61 ± 0.26	0.0907	0.0999	4.71
Tarda	110 V	167.7	16.6547	72.49 ± 0.22	0.0823	0.0906	4.73
Tarda	100	124.3	16.0960	70.06 ± 0.14	0.0821	0.0894	4.91
Tarda^g^	100	99.5	11.0047	47.90 ± 0.08	0.0634	0.0683	5.36
Tarda	VRT	98.6	15.1008	49.34 ± 0.14	0.0671	0.0719	5.29
Tarda	NH	59.5	7.7325	33.67 ± 0.25	0.0531	0.0507	5.64
Ivuna	100	106.6	4.2185	18.36 ± 0.10	0.0828	0.0840	15.56
Orgueil	100	95.5	3.4934	15.21 ± 0.09	0.0643	0.0652	15.11
Aguas Zarcas	100	134.1	3.7734	16.42 ± 0.06	0.0588	0.0613	14.40
Aguas Zarcas	NH	131.4	2.3602	10.27 ± 0.07	0.0407	0.0263	15.10
Murchison	100	99.4	3.1727	13.81 ± 0.08	0.0524	0.0545	14.58
Allende	100	64.9	0.2775	1.21 ± 0.01	0.0074	0.0082	29.0

**g**—exposed to 100% RH at 32 °C for 24 h forming gypsum, then outgassed under flowing N_2_ at 100 °C. **NH**—sample not outgassed at 100 °C for 24 h. **VRT**—outgassed at room temperature under vacuum for 24 h. **100**—outgassed under flowing dry N_2_ at 100 °C for 24 h. **110 V**—outgassed under vacuum at 100 °C for 24 h. **250**—outgassed under flowing dry N_2_ at 250 °C for 24 h. **1**—BJH Adsorption cumulative volume of pores between 1.7 nm and 300.0 nm width. **2**—BJH Desorption cumulative volume of pores between 1.7 nm and 300.0 nm width. **3**—BJH Adsorption average pore width (4 V/A).

The *S*_BET_ values of the clay-rich meteorites measured here are within the range of terrestrial argillaceous rocks and clays^1,17–20,34^. For example, *S*_BET_ for The Clay Minerals Society source clays measured by N_2_ BET range from 12.1 to 173 m^2^/g^17^, though the three natural smectites are 22.7 to 65.2 m^2^/g. The *S*_BET_ values for Orgueil and Ivuna are significantly less than the mineralogically similar Tarda despite the similar bulk mineralogy. The value measured for Orgueil-100 of 15.21 m^2^/g is half that previously reported^22^, despite the similar outgassing conditions. This difference may be the result of the varied curatorial histories of samples since its fall in 1864, however, the value measured here is still within the range for smectite-rich argillaceous rocks. In addition, the S_BET_ data for Tarda acquired after different pretreatment and outgassing treatments vary by over a factor of × 2 for the sample run directly from the dry N_2_ chamber to the one outgassed at 250 °C under flowing N_2_.

Information on the pore-size distribution and average pore size is gained from the isotherm data by employing the t-Plot method using a Harkins and Jura thickness equation and BJH analyses with Halsey-Faas correction to derive the pore data. The single-point cumulative volume of pores in the 1.7 nm to 300.0 nm width range (V_ad_) calculated from the adsorption data for the clay-rich meteorites ranges from 0.0524 to 0.0907 cm^3^/g (Table 1). The same calculated from the desorption data (V_ds_) is of a similar magnitude, though in general a few percent larger (Table 1). Despite Tarda-110 V having a significantly higher S_BET_ than Ivuna, their V_ad_ values are close. In general, V_ad_ for the serpentine-rich Aguas Zarcas and Murchison are lower than the smectite-rich meteorites, with the anhydrous Allende showing the lowest V_ad_ value. However, there are significant differences in their < 200 nm pore-size distributions derived from the N_2_ BET data with the Halsey Faas correction calculated from the adsorption isotherms.

**Figure 2:**
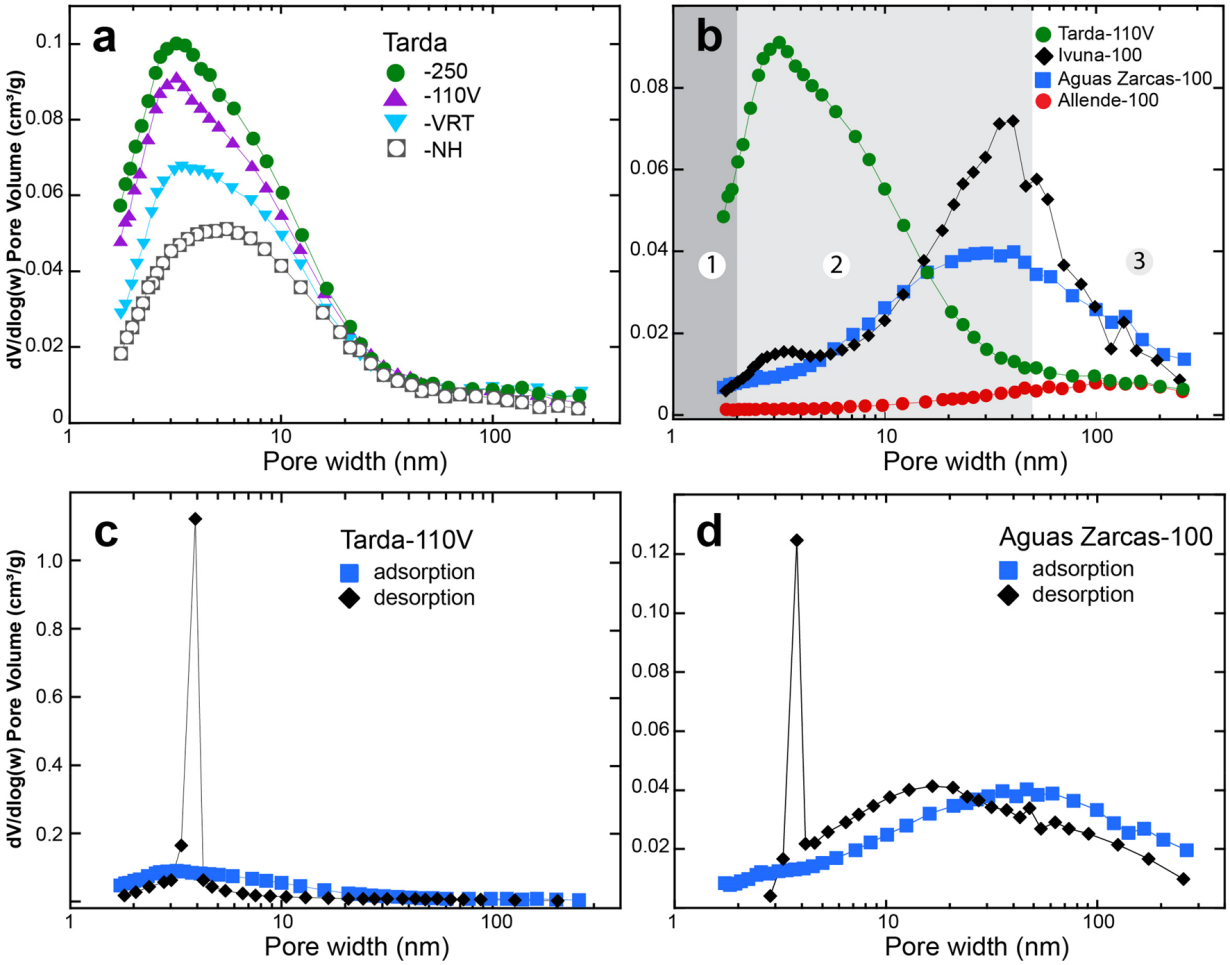
Comparison of the logarithmic differential pore-volume distribution, dV/d (log(w)) versus pore width calculated from the N_2_ BET data with the Halsey Faas correction. (**a**) Pore-volume distribution for Tarda after different outgassing pretreatments derived from the adsorption isotherms. (**b**) Comparison of the pore-volume distribution for Tarda, Ivuna, Aguas Zarcas, and Allende. The regions 1 to 3 correspond to the micro-, meso- and macropore volumes, respectively. (**c**) Pore-volume distribution for Tarda-110 V. (**d**) Pore-volume distribution for Aguas Zarcas. Outgassing conditions are: -250, heating to 250 °C under flowing N_2_ for 24 h; -110 V, heating to 110 °C under vacuum for 24 h; -100, heating to 100 °C under flowing N_2_ for 24 h; -VRT, kept under vacuum at room temperature for 24 h; and -NH, sample not outgassed and run directly from the N_2_ storage.

A commonly used method to display the pore-size distribution is through the plot of the logarithmic differential pore-volume distribution, dV/d (log(w)), versus pore width^1,13^. Here, the area under the curve in any pore diameter range yields the volume of pores in that range. These plots derived for the adsorption branch of the isotherms show that Tarda has significant mesoporosity, with a maximum near the upper boundary with the micropore range and a broad tail into the macropore region (Fig. 2a). The profile maximum becomes narrower and more intense from Tarda NH → VRT → 100/110 V → 250. In contrast, the mineralogically similar Orgueil and Ivuna have a bimodal pore-size distribution with a major maximum near 40 nm and a minor peak around 3 nm (Fig. 2b and S9). The profiles for Aguas Zarcas and Murchison show a less-pronounced 3-nm peak and broader maximum that straddles the meso-macropore boundary (Fig. 2b and S10). The anhydrous Allende lacks significant microporosity and has a broad maximum near 100 nm (Fig. 2b). While the application of Kelvin equation-based procedures, such as the BJH method, can with some pore geometries underestimate the pore size, e.g.^10^, the plots of pore width versus the log differential pore size for the meteorites provide a semi-quantitative view of the pore-size distribution.

In certain pore geometries, the desorption isotherm can provide a more accurate representation of the pore geometry as it is thought that the desorption process is in thermodynamic equilibrium between the liquid adsorbed phase in the pores and the external gaseous phase. However, the smectite and serpentine-rich meteorites studied here all show the sudden abrupt closure of the desorption branch to the adsorption branch of the isotherm near p/p^0^ ~ 0.4. The plots of pore width versus the log differential pore size determined from the desorption isotherms of the clay-rich meteorites all show a sharp spike at 3.8 nm (Fig. 2c, d). This spike is caused by rapid desorption during evaporation from the pore neck and the pore body involving cavitation and the growth of vapor bubbles in the metastable condensed fluid and is evidence for pore restriction smaller than ~ 5 to 6 nm for N_2_ at 77 K ^11,12^.

The peak in the micropore-mesopore boundary region in the pore-size distribution from smectite-rich rocks is attributed to intra- and inter-tachoid porosity^1,35^. Tachoids are 2- to 50-nm-sized aggregates with turbostratic stacking of the phyllosilicate TOT plates. Tarda is smectite-rich, and a maximum in the micropore region is consistent with porosity between the tachoids. The rapid decrease in dV/dlog(w) with the increase in pore size (Fig. 2a) suggests a decreasing density of pores in the 50- to 100-nm-size range between the tachoids, whereas Orgueil, Ivuna, Aguas Zarcas, and Murchison possess significant inter-tachoid and intra-aggregate porosity. For example, HRTEM images of Orgueil show a highly disordered submicron mélange of interpenetrating platy, curved, and poorly crystalline phyllosilicates and ferrihydrite together hosting nanometer-sized sulfides^29^, with abundant sites for intra-tachoid porosity. More recently, HRTEM images from Ryugu C1-like material show similar fine-scale phyllosilicate complexity^36^. In both the Orgueil and Ryugu material the nanoscale tachoid nature of the matrix is evident in the HRTEM images. In contrast, the matrices of the CM2 chondrites show regions of more coarsely crystalline phyllosilicates commonly with platy and polygonal morphologies as well as regions with submicron tissue-like aggregates^37–40^.

### Characterizing the adsorbed water

The increase in S_BET_ from Tarda NH → VRT → 100/110 V → 250 is consistent with the removal of gases from the physisorbed sites. Information on the identity of the physisorbed gases can be measured by thermal gravimetric analysis (TG) combined with a mass spectrometric evolved-gas analysis system (MSEGA). MSEGA detects evolved gases that have distinct ion mass-to-charge ratios (m/z). These methods are used to provide information on the H_2_O and OH^-^ content of the phyllosilicates and other H-bearing in the CC meteorites^22,25,41,42^. The thermal analysis was recently described from Aguas Zarcas^25^ so the focus here is on Tarda. Although samples were heated to 1000 °C, the primary temperature range of interest here is below 300 °C, which is within the range that the samples were heated during the outgassing for the N_2_ BET analyses and lower than the dehydroxylation temperatures of the phyllosilicates.

**Figure 3:**
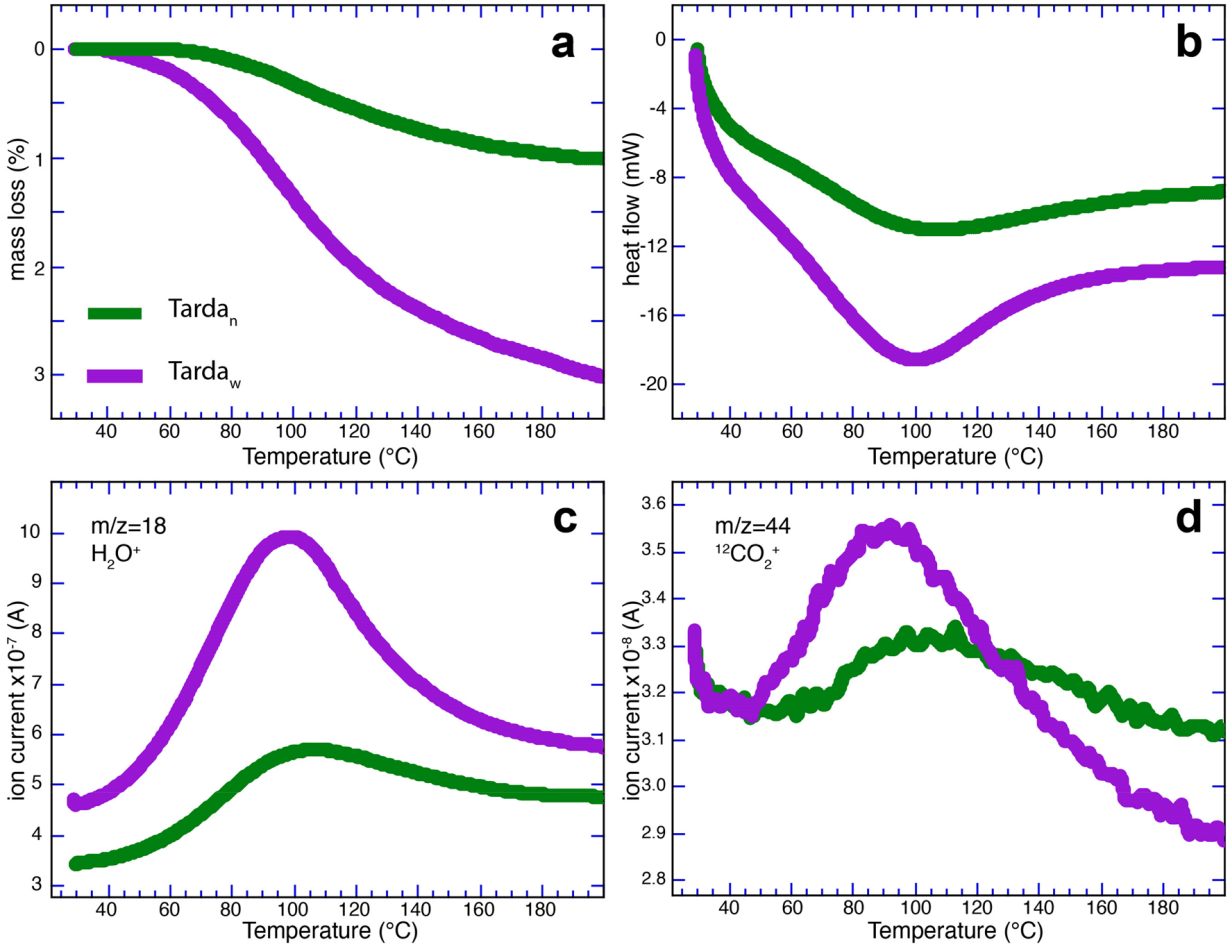
TG-DSC-MSEGA for Tarda before and after artificial weathering. (**a**) TG, (**b**) DSC, (**c**) MSEGA for m/z = 18 (H_2_O), and (**d**) MSEGA for m/z = 44 (CO_2_) curves for Tarda curated under nitrogen (green curves) and artificially weathered with water (purple curves). Data are shown for the low-temperature region up to 200 °C. The complete data to 1000 °C are shown in Figs. S11 to S15.

Two Tarda samples were analyzed. The first is a fresh powder curated under a dry N_2_ atmosphere – Tarda_N_. The second, called Tarda_W_, was a powder mixed with distilled water and allowed to dry at room temperature under ~ 34% RH. The TG mass losses for Tarda_N_ and Tarda_W_ heated to 1000 °C are 16.6 and 19.4%, respectively (Figs. 3, S11, S12). Their TG, DSC, and MSEGA profiles are broadly similar (Figs. S11–S15), though there are significant differences below 200 °C (Fig. 3). The DTG curves show three prominent features near 100°, 510°, and 760° C, corresponding to the significant rates of change in the TG mass loss curve. The first mass loss step for Tarda_N_ and Tarda_W_ between 60 and 200 °C are Δm −1.011% and −3.013%, respectively (Figs. 3a, S11, S12). This first mass loss step corresponds to the loss of adsorbed water and water intercalated with the smectite clays. The 100 °C peak in the DTG curve corresponds to the endothermic peak in the DSC curve (Fig. S14).

The identity of the gas species evolved corresponding to specific regions of the TG loss curve is revealed by the MSEGA data. A wide range of ion species evolved during heating and most of the ion signals for Tarda_N_ and Tarda_W_ are similar over the 1000 °C range (Fig. S15). However, below 300 °C there are yield differences for m/z = 18, 30, and 44. The most abundant gas released below 300 °C has m/z = 18 corresponding to H_2_O and its signal is significantly more intense for Tarda_W_. The signal for m/z = 44, corresponding to CO_2_, is more intense for Tarda_W_ with a maximum near 90 °C (Fig. 3d). Below ~ 200 °C, there is little evidence from the MSEGA data for the evolution of organic compounds. For example, significant signals for m/z = 15 (CH_3_^+^, methyl derivatives) and 26 (C_2_H_2_^+^ from aromatic hydrocarbons) are absent at this low-temperature range (Fig. S15). However, the signal for m/z = 30 shows two maxima below 300 °C at 100 °C and 200 °C for Tarda_W_, whereas Tarda_N_ only shows a weak maximum at 200 °C (Fig. S16). This peak can have several origins, including CH_2_O^+^ and C_2_H_6_^+^.

The mass loss for Tarda_W_ below 200 °C of 3.01 wt% is at the lower end for the two published values of 3.7 and 7 wt%^43,44^. This mass-loss range is typical for smectite-rich type 1 meteorites of ~ 5 to ~ 10 wt%^21,22,41,42^. However, samples dried under flowing He for 24 h show a mass loss of 1.011 wt% (Fig. 3a, S11), whereas the artificially weathered sample has a mass loss of 3.013 wt % after being dried under laboratory conditions with ~ 35% RH (Fig. 3a, S12).

### Mineralogical and physical effects of high surface area

The high S_BET_ of the clay-rich meteorites and the nanoscale interconnected porosity allow atmospheric water vapor to impinge upon the bulk of the matrix mineral network. In addition, the terrestrial alteration of Tarda and the CI chondrites is accentuated by the ability of their abundant matrix smectite to intercalate water^22,45^. While the S_BET_ derived from N_2_ BET gives a measure of the N_2_ accessible surface area, this gas does not probe the interlayer adsorption sites of the smectite. For example, the surface area of Orgueil measured by the BET using N_2_ is 30.2 m^2^/g and 165.8 m^2^/g using H_2_O^22^. Water intercalates around the interlayer cations between stacked 2:1 layers of the smectite clays. The intercalation of water by the smectite group minerals has been extensively studied^34,45–50^. The degree of intercalation depends on the clay's elemental properties, particularly the type of interlayer cation, and p/p^0^ of the surrounding water vapor^45^. Increasing p/p^0^ causes interlayer adsorption with a stepwise increase in the d_001_ spacing of smectite. For example, the d_001_ of Ca montmorillonite increases from 9.6 to 10.7 Å for the fully dehydrated form, to 11.8–12.9 Å, 14.5–15.8 Å, to 18.0–19.5 Å, for the mono-, bi- and tri-hydrate, respectively^45,48^. In contrast, the serpentine 001 reflection from Aguas Zarcas and Murchison is not affected by the water uptake.

**Figure 4:**
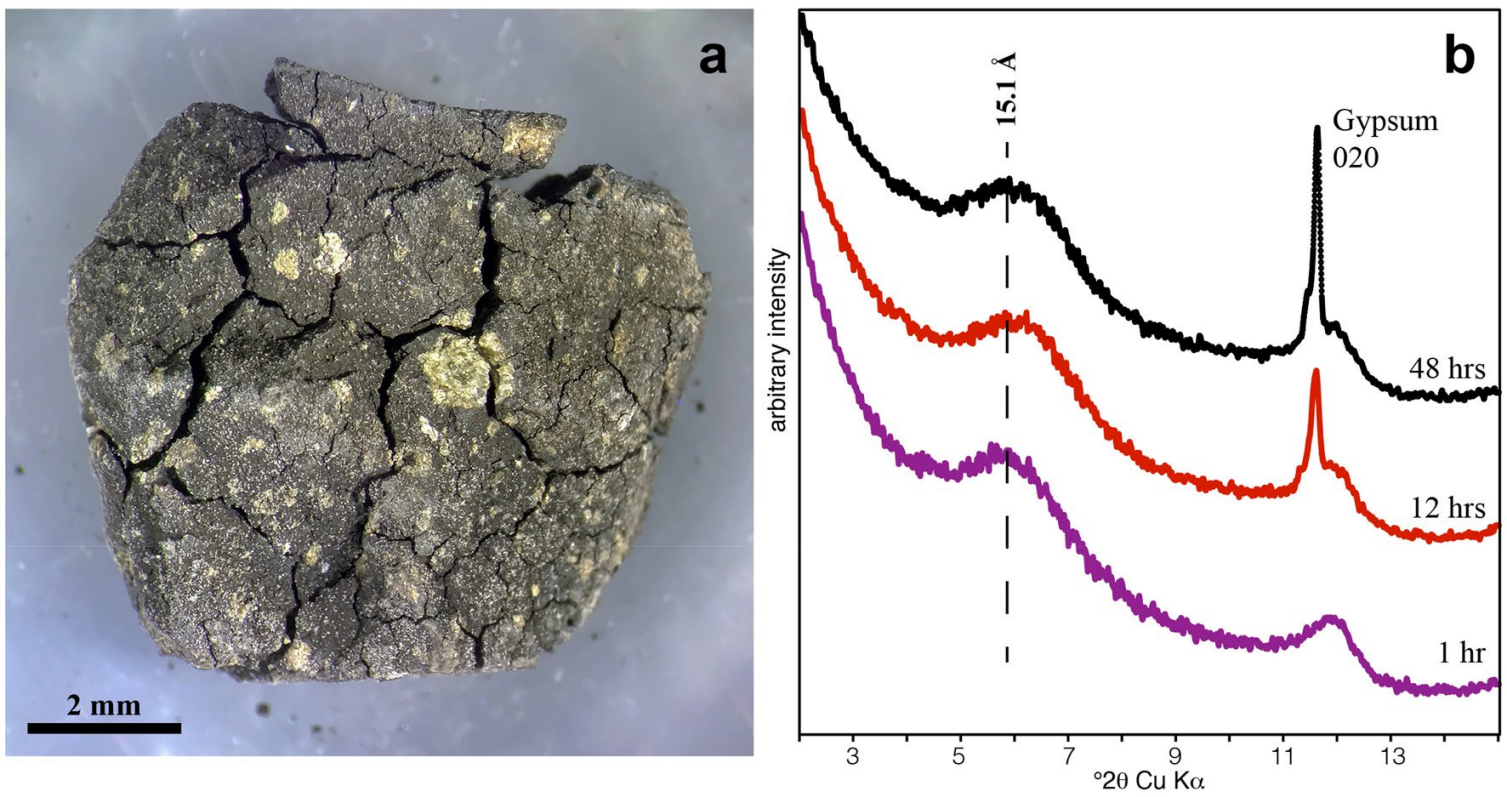
(**a**) Photograph of a Tarda fragment after 14 days at 32 °C and 100% RH. The sample shows extensive cracking and formation of gypsum (small white dots). (**b**) Powder XRD pattern from the same Tarda sample after drying (1 h) and after 12 and 48 h at 100% RH showing the formation of gypsum. The patterns have been shifted along the y-axis for clarity.

Tarda swells and cracks under high humidity and cycles of higher and lower humidity cause spalling of the fragments (Figs. 4a, S17). This cracking is accompanied by the growth of gypsum on the fragments within a few days under high relative humidity. Gypsum growth is instituated by the high surface area that forms the interconnected nanoporous sponge-like network of nano-sized pathways. The rapidity of gypsum formation was evident from the powder XRD, which shows a prominent 020 reflection after only 12 h at 100% RH (Fig. 4b). The formation of the gypsum is an irreversible mineralogical change that increases the mass of the sample and decreases the surface area. For example, the S_BET_ of the gypsum-rich Tarda (Tarda^g^) was significantly lower than Tarda-110 V (Table 1).

Tarda shows an extreme example of terrestrial alteration by rapidly slaking in water (Supplementary Movie1) and other polar liquids. Slaking is shown by many clay-rich soils^51^. From a practical point of view, this slaking is problematic as many laboratory sample preparation techniques, such as cutting, grinding, and polishing use polar liquids, most commonly water, ethylene glycol, alcohols, or acetone as a lubricant. This behavior is not unique to Tarda. For example, in 1834 Berzelius said that in the presence of water the CI1 chondrite Alais “… zerfällt er nach einigen Augenblicken su einem graugrünen Brei … [… transforms within a few moments into a grey-green paste …].”^52^. This extreme reaction to polar liquids has implications for curation as well as sample preparation for analytical studies. The propensity of the smectite-rich meteorites, and to a certain degree those that are serpentine-rich, to disintegrate with polar liquids requires alternative cutting and polishing methods^53^.

### Implications

The S_BET_ of Tarda increases with the following outgassing conditions NH → VRT → 100/110 V → 250 (Table 1) reflecting the increasing removal of adsorbed gases from the N_2_-accessible surface area. The TG measurements demonstrate the quantity of atmospherically adsorbed gases through mass loss and the MSEGA show that it is primarily H_2_O with minor CO_2_. It is likely that the S_BET_ measured for the Tarda-250 and Tarda-110 V (Table 1) is within the range of the pre-atmospheric material. However, the S_BET_ for these outgassed samples may still be lower than its S_BET_ before entering the Earth’s atmosphere, because the nano-scale porosity is so rapidly blocked and modified during terrestrial residence. This blocking was demonstrated by subjecting Tarda to 100% RH at 32 °C for 24 h after which the S_BET_ was reduced to 47.9 m^2^/g. This reduction is accompanied by the growth of gypsum on and within the fragments. Furthermore, the surface area adsorption capacity of smectite-rich meteorites with H_2_O will be significantly higher than the S_BET_ determined with N_2_ BET, as nitrogen does not probe the interlayer space of the 2:1 layer-structured clay.

In comparison with Tarda, the Ivuna, and Orgueil (CI1) and Aguas Zarcas and Murchison (CM2) meteorites have significantly lower S_BET_ of ~ 14 to 19 m^2^/g. Their pores have a bimodal size distribution with a lower number of ~ 3-nm-sized pores and a broader size distribution around 40 nm. The ease with which the S_BET_ of Tarda is reduced by even short exposure to Earth’s atmosphere may explain the significantly lower surface area measured for the mineralogically similar Ivuna and Orgueil. Ivuna and Orgueil have had relatively long residence times on Earth, i.e., 1938 and 1864, respectively, which led to the formation of pore-blocking secondary minerals. In contrast, the serpentine-rich meteorites studied here have similar S_BET_ values even though Murchison landed on Earth in 1969 and spent decades stored under atmospheric conditions. In contrast, the Aguas Zarcas studied here was collected within a day of falling on Earth and stored under a dry N_2_ atmosphere.

From a practical point of view, the rate and ease with which these clay-rich astromaterials adsorb atmospheric gases, but not limited to H_2_O, implores the need for curation in a stable atmosphere with a constant low relative humidity. The revelation that smectite- and serpentine-rich astromaterials possess an intrinsic high-surface area with a nanoporous network has implications for other physical properties including sound speed, thermal conductivity, and compressive strength which depend on the structural distribution within the material. These astromaterial properties govern, for instance, the resistance of boulders on the surface of an asteroid to hypervelocity impacts and thermal cycling on their parent body. They also govern how, and how deep, a meteor comes apart during atmospheric entry. The measurement of porosity is probably the easiest way of probing the nano-scale structure of astromaterials, complementary to measurements of crush curves and other failure mechanisms.

The TG and BET data for Tarda suggest that the quantity of molecular water intercalated with the clays before arrival on Earth is minor. This postulation is based on the fact that before its arrival on Earth, the Tarda meteoroid was subjected to the ultrahigh vacuum of space, with surface temperatures between 100 and 200 °C near 1 a.u. These conditions would lead to an N_2_-accessible surface area similar to the sample heated to 110 °C under a high vacuum. This result is corroborated by the low mass loss of ~ 0.6 wt% below 200 °C for the mineralogically similar samples from asteroid Ryugu^54^.

The N_2_ BET analysis of the clay-rich meteorites reveals scale-dependent aspects of their interiors. The N_2_-accessible surface area, which is formed by the interconnected mesoporosity, constitutes a sponge-like network with nano-sized pathways onto which atmospheric gases can physisorb. Further, water vapor can also intercalate into the interlayer sites of the smectite clays thus significantly increasing their adsorption characteristics. The Tarda results measured here have implications for the recently returned asteroid samples from Ryugu and Bennu. The asteroid-returned samples have mineralogical and structural similarities to Tarda and will likely have the similarly high surface area and fine-scale, interconnected nanoporosity.

## Experimental section

### Meteorites and curatorial conditions

All the meteorites studied here are curated under a dry N_2_ atmosphere in the Carleton B Moore Meteorite Collection in the Buseck Center for Meteorite Studies (BCMS) at Arizona State University (ASU). The following samples were studied—Tarda (ASU#2149), Ivuna (ASU#856), Orgueil (ASU#222), Aguas Zarcas (ASU#2121), Murchison (ASU#828), and Allende (ASU#818). Aguas Zarcas fell in Costa Rica on the 23^rd^ of April 2019. Samples were collected and returned to the BCMS by Michael Farmer within one week of the fall and curated under a dry N_2_ atmosphere. Tarda fell in southern Morocco on the 25^th^ of August 2020 and samples were collected within a few days of the fall and acquired by BCMS in September 2020 where they are curated under a dry N_2_ atmosphere. Neither Aguas Zarcas nor the Tarda samples received by BCMS saw significant moisture, other than atmospheric air, before curation under the dry N_2_ atmosphere. In addition, selected comparative measurements were undertaken on Murchison (CM2), Ivuna (CI1), and Orgueil (CI1). Murchison fell over Murchison, Australia on the 28^th^ of September 1969. Ivuna fell near the western shore of Lake Rukwa in Tanzania on the 16^th^ of December 1938, and Orgueil fell near Montauban in southwestern France on the 14^th^ of May 1864. Humidity in air near 0% was achieved by placing the samples in a bell jar which contained an open container of the drying agent P_2_O_5_. Laboratory temperature and humidity were measured with an Onset HOBO® U12 datalogger. Relative humidity of 100% was achieved by placing the sample in a sealed container containing water. Sample masses were measured with a Mettler Toledo AR201 analytical balance with repeatability (sd) of 0.04 mg and readability of 0.01 mg.

### BET N_2_ analysis

Adsorption/desorption isotherms were measured under N_2_ at −195.8 °C on a Micromeritics® TriStar II Plus surface area and porosity analyzer. The meteorites were characterized by applying the BET N_2_ sorption method. Data was analyzed using the t-Plot method assuming a Harkins and Jura thickness equation and BJH analyses with Halsey-Faas correction to derive the pore data. Measurements were made on Tarda (C2-ung), Ivuna (CI1), Orgueil (CI1), Aguas Zarcas (CM2), Murchison (CM2), and for comparison the anhydrous carbonaceous chondrite Allende (CV). Sample sizes for the BET measurements were on the order of 0.5 to 0.8 g. Samples were run as mm-sized fragments. In this paper, the outgassing pretreatment is listed after the meteorite name: Tarda-100 sample outgassed at 100 °C under flowing N_2_ for 24 h; Tarda-110 V outgassed for 24 h at 110 °C with a final vacuum of 5.6E-6 torr; after prolonged storage under a dry N_2_ atmosphere (–NH); after being held under a vacuum for 24 h at room temperature (–VRT); and, after heating in the presence of flowing dry N_2_ at 250 °C for 24 h (−250). Vacuum desorption was accomplished by heating the tube in a mantle while attached to a glass, high-vacuum manifold evacuated through a liquid nitrogen trap with an oil diffusion pump backed by an oil-sealed rotary vane pump. The sample tube was attached by a Swagelok Ultra-Torr fitting to a stopcock that was then attached to the manifold so that an inert atmosphere could be maintained while the tube was transferred to the glove box. The BET measurements were acquired over the relative pressure range p/p^0^ (p is the actual gas pressure and p^0^ is the vapor pressure of the adsorbing gas) of ~ 0 to 0.99, which corresponds to the absolute pressure of ~ 0.8 to 730 mmHg. A value of 0.1620 nm^2^ was used as the molecular cross-sectional area for N_2_. The N_2_ BET data are listed in the data document^33^.

### Powder X-ray diffraction

Powder XRD patterns were acquired with a Rigaku MiniFlex 600 diffractometer. This diffractometer is operated with Cu*Kα* radiation and is equipped with a post-diffraction graphite monochromator and automatic divergence slit system. Data were acquired from 2° to 65° 2θ at 0.02° steps, and 30 to 60 s/step. XRD samples were prepared from an ~ 1- to 2-mm-sized fragment, which weighed ~ 10 mg. The chips were crushed and lightly ground to a fine powder and mixed with a few milliliters of dry methanol. The resulting slurry was pipetted and spread into a thin, smooth film on a low-background, single-crystal, quartz plate. This slurry was dried rapidly (~ 5 s) under flowing warm air forming a thin film.

### TG–DTA/DSC

Thermal measurements were performed on a Setaram LabsysEvo (Lyon, France) TG–DTA/DSC system, in a flowing (60 mL/min) purging gas atmosphere [99.9999% purity He /DTA/, 99.999% purity Ar /DSC/ and 99.999% purity synthetic air (20% O_2_ in N_2_) /DSC/ atmospheres]. The sample was weighed into a 100 μL Al_2_O_3_ crucible (the reference crucible was empty) and heated from 25 to 1000 °C with a heating rate of 10 °C/min. The obtained data was baseline corrected and further processed with the thermoanalyzer’s processing software (Calisto Processing, ver. 2.092). The thermal analyzer (both the temperature scale and calorimetric sensitivity) was calibrated by a multipoint calibration method, in which seven different certified reference materials (CRMs) were used to cover the thermal analyzer’s entire operating temperature range.

### TG-DSC-MSEGA

Thermal measurements were performed on a Setaram LabsysEvo (Lyon, France) TG-DSC system, in a flowing (90 mL/min) helium gas (99.9999% purity) atmosphere. The sample was weighed directly into a 100 μL Al_2_O_3_ crucible (the reference cell was empty) and was heated from 25 to 1000 °C with a heating rate of 20 °C/min. The obtained data was baseline corrected and further processed with the thermoanalyzer’s processing software (Calisto Processing, ver. 2.092). The thermal analyzer (both the temperature scale and calorimetric sensitivity) was calibrated by a multipoint calibration method, in which seven different certified reference materials (CRMs) were used to cover the thermal analyzer’s entire operating temperature range. In parallel with the thermal measurements, the analysis of evolved gases/volatiles was performed on a Pfeiffer Vacuum Omni Star™ mass spectrometric evolved gas analysis system (MS-EGA) connected to the above-mentioned thermal analyzer. The gas splitter was thermostated to 230 °C, while the transfer line to the mass spectrometer was thermostated to 220 °C. The temperature of the mass spectrometer gas inlet was programmed to 120 °C. The measurements were done in SEM Bargraph Cycles acquisition mode, where the m/z interval of 11–130 was continuously scanned with a speed of 50 ms/amu. The spectrometer was operated in electron impact mode. The “artificially weathered” Tarda was prepared by mixing 33 mg of dried, as received, powder was 100 mL of ultrapure water and allowed to evaporate to dryness. TG-DSC-MSEGA data were acquired from the dried powder.

### Supplementary Information

Supplementary Information 1.

Supplementary Video 1.

## Data Availability

Further data and plots are provided in the attached supplementary files. The BET, TG, and MSEGA data are available for download at https://data.mendeley.com/datasets/fr95xpvjt7/1. 10.17632/fr95xpvjt7.1.
