# Supplementary material for: High surface area and interconnected nanoporosity of clay-rich astromaterials
Source: Sci Rep. 2024 May 6;14:10358. doi: 10.1038/s41598-024-61114-2 (PMC11074322; doi:10.1038/s41598-024-61114-2)
Supplement: Supplementary file 1 — Supplementary Information 1. [file 41598_2024_61114_MOESM1_ESM.docx]

**Supplementary Data**

**The breathing of clay-rich astromaterials on Earth**

Laurence A.J. Garvie^1,2^, László Trif^3^, Desiree Cotto-Figueroa^4^, Erik Asphaug^5^, and Christian G. Hoover^6^

**Bulk mineralogical characterization.**

The powder XRD patterns of Tarda, Ivuna, and Orgueil are dominated by reflections from smectite, serpentine, and interstratified serpentine/smectite (Fig. S1), with significant magnetite, pyrrhotite, carbonates, and lesser forsterite ^1-4^. The overall shape of the phyllosilicate region of the XRD patterns of Tarda, Ivuna, and Orgueil are similar suggesting similar bulk matrix mineralogies. The breadth of their phyllosilicate reflections is consistent with grains that are on average fine-grained, poorly crystalline, with turbostratic stacking, interstratified, or a combination thereof ^5,6^. For example, HRTEM images of Orgueil show that the phyllosilicates occur as poorly crystalline, fine crystallites intimately associated with ferrihydrite, and clusters of coarser phyllosilicates relatively free of ferrihydrite ^7^. Powder XRD patterns from Aguas Zarcas and Murchison are dominated by reflections from serpentine group minerals, with locally abundant ferrotochilinite and 1:1 regularly interstratified ferrotochilinite/cronstedtite (Fig. S2), anhydrous silicates, calcite, pentlandite, pyrrhotite, and minor phases ^8-10^.

**Supplementary References cited**

1 Brearley, A. J. in *Lunar and Planetary Science Conference, volume 23, page 153.*

2 Garvie, L. A. J. & Trif, L. in *52nd Lunar and Planetary Science Conference.* 2446.

3 King, A. J., Schofield, P. F., Howard, K. T. & Russell, S. S. Modal mineralogy of CI and CI-like chondrites by X-ray diffraction. *Geochim. Cosmochim. Acta* **165**, 148-160 (2015). <https://doi.org:10.1016/j.gca.2015.05.038>

4 Yesiltas, M. *et al.* Compositional and spectroscopic investigation of three ungrouped carbonaceous chondrites. *Meteoritics & Planetary Science* **57**, 1665-1687 (2022).

5 Brindley, G. W. in *Crystal Structures of Clay Minerals and their X-ray Identification* (eds G.W. Brindley & G. Brown) 125-196 (Mineralogical Society, 1980).

6 Reynolds, R. C. in *Crystal Structures of Clay Minerals and their X-ray Identification* (eds G.W. Brindley & G. Brown) 249-304 (Mineralogical Society, 1980).

7 Tomeoka, K. & Buseck, P. R. Matrix mineralogy of the Orgueil CI carbonaceous chondrtie. *Geochim. Cosmochim. Acta* **52**, 1627-1640 (1988). <https://doi.org:10.1016/0016-7037(88)90231-1>

8 Fuchs, L. H., Olsen, E. & Jensen, K. J. Mineralogy, mineral-chemistry, and composition of the Murchison (C2) meteorite. *Smithsonian Contributions to the Earth Sciences* **10**, 39 (1973).

9 Garvie, L. A. J. Mineralogy of the 2019 Aguas Zarcas (CM2) carbonaceous chondrite meteorite fall. *American Mineralogist* **106**, 1900-1916 (2021).

10 Kerraouch, I. *et al.* The polymict carbonaceous breccia Aguas Zarcas: A potential analog to samples being returned by the OSIRIS‐REx and Hayabusa2 missions. *Meteoritics & Planetary Science* **56**, 277-310 (2021).


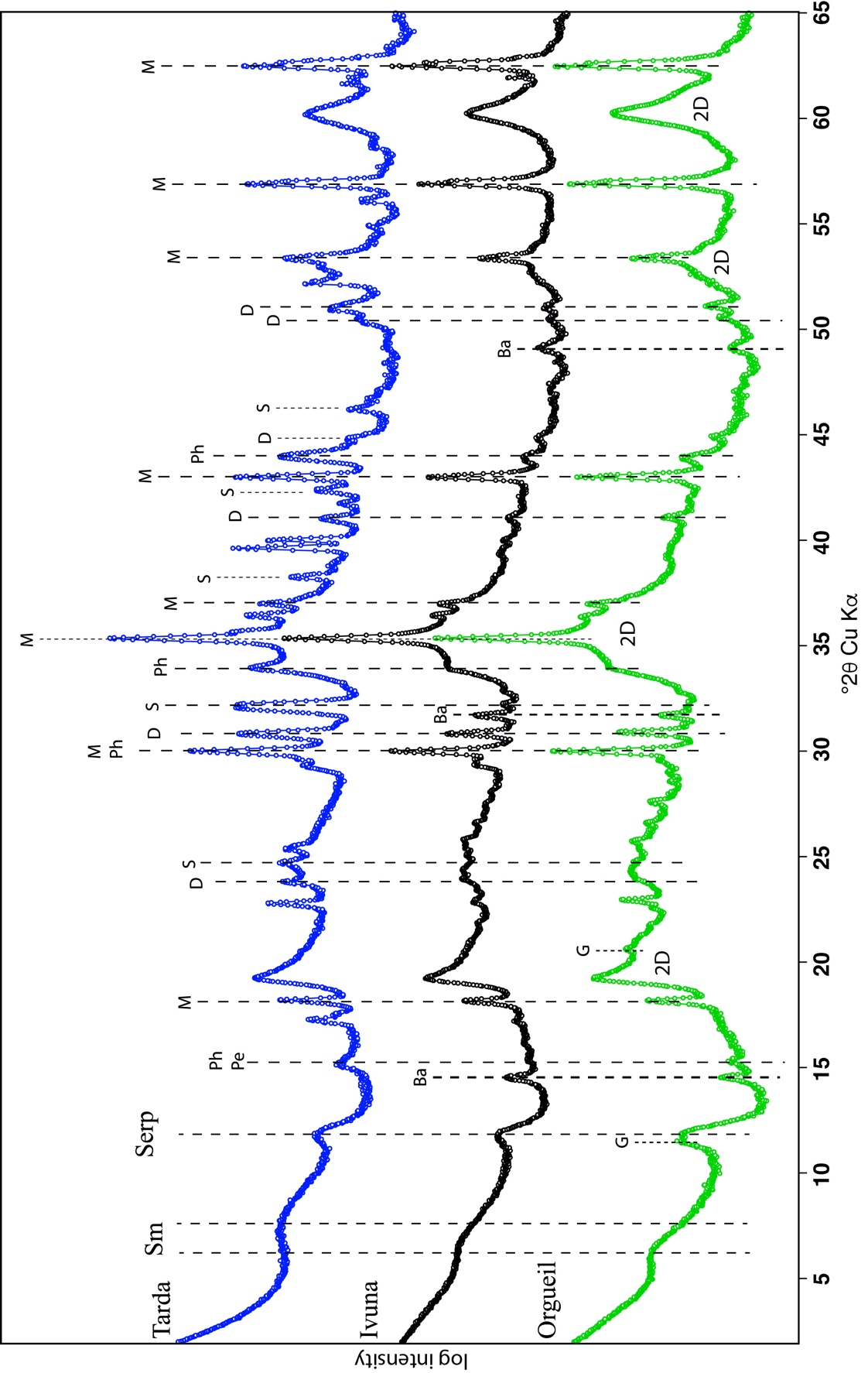


**Figure S1.** Powder X-ray diffraction patterns for Tarda, Ivuna, and Orgueil are shown on a log intensity scale and shifted along the y-axis for clarity. Phases marked are Sm – smectite, Serp – serpentine, G – gypsum, Ba – bassanite, Ph – pyrrhotite, Pe – pentlandite, M – magnetite, D – dolomite, S – siderite. 2D – indicates the broad phyllosilicate two-dimensional diffraction bands.


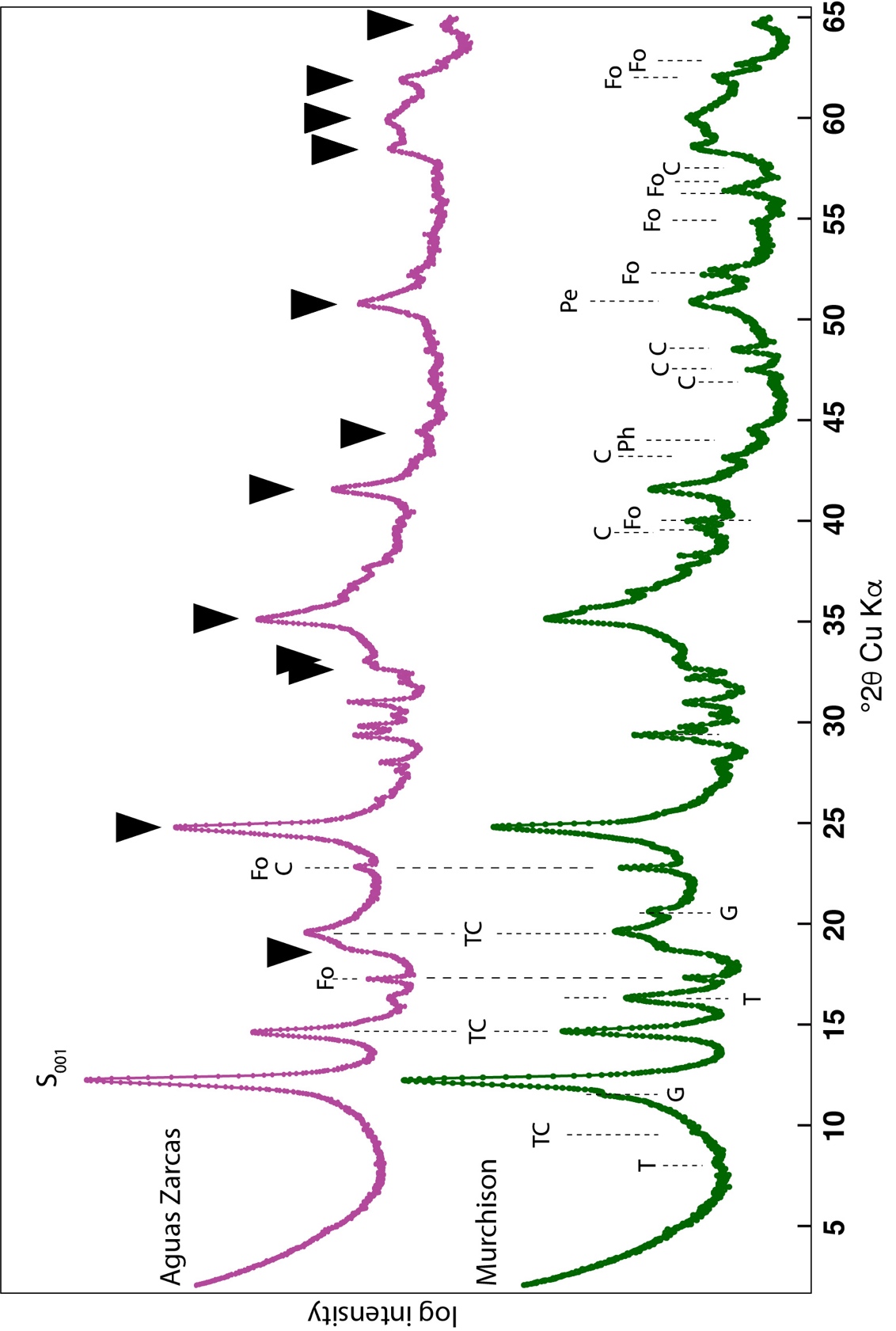


**Figure S2.** Powder X-ray diffraction patterns for Aguas Zarcas and Murchison are shown on a log intensity scale and shifted along the y-axis for clarity. The Aguas Zarcas is from a chondrule-poor fragment (ASU#2121_6) and Murchison is from stone ASU#828_3. Phases marked S_001_ – serpentine 001 reflection, Pe – pentlandite, Ph – pyrrhotite, T – ferrotochilinite, TC – regularly interstratified ferrotochilinite/cronstedtite, Fo – forsterite, C – calcite, D – dolomite. Serpentine reflections indicated by an 🡻.


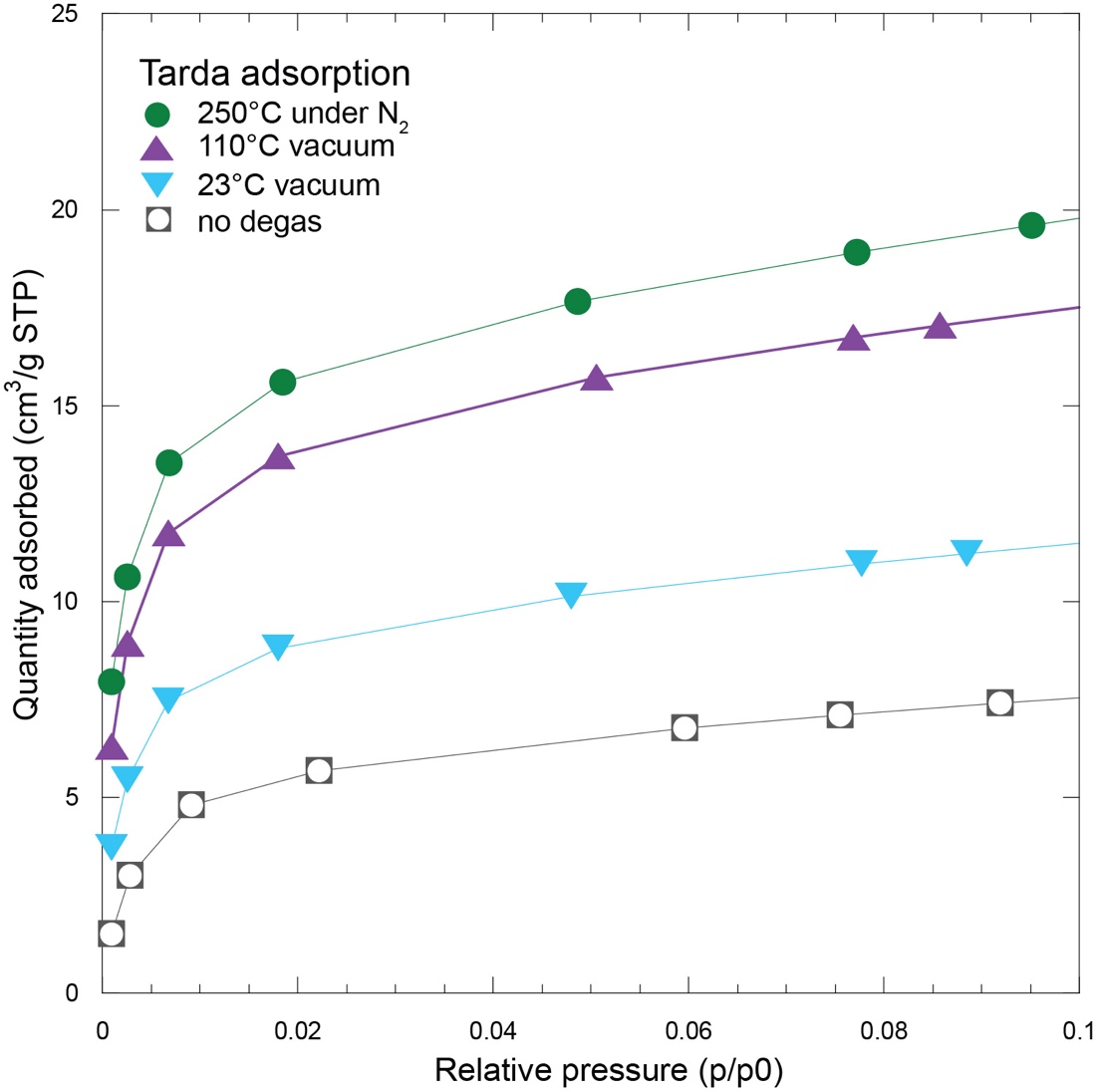


**Figure S3.** N_2_ BET isotherm acquired at 77 K for Tarda in the 0<p/p^0^<0.1 relative pressure range.


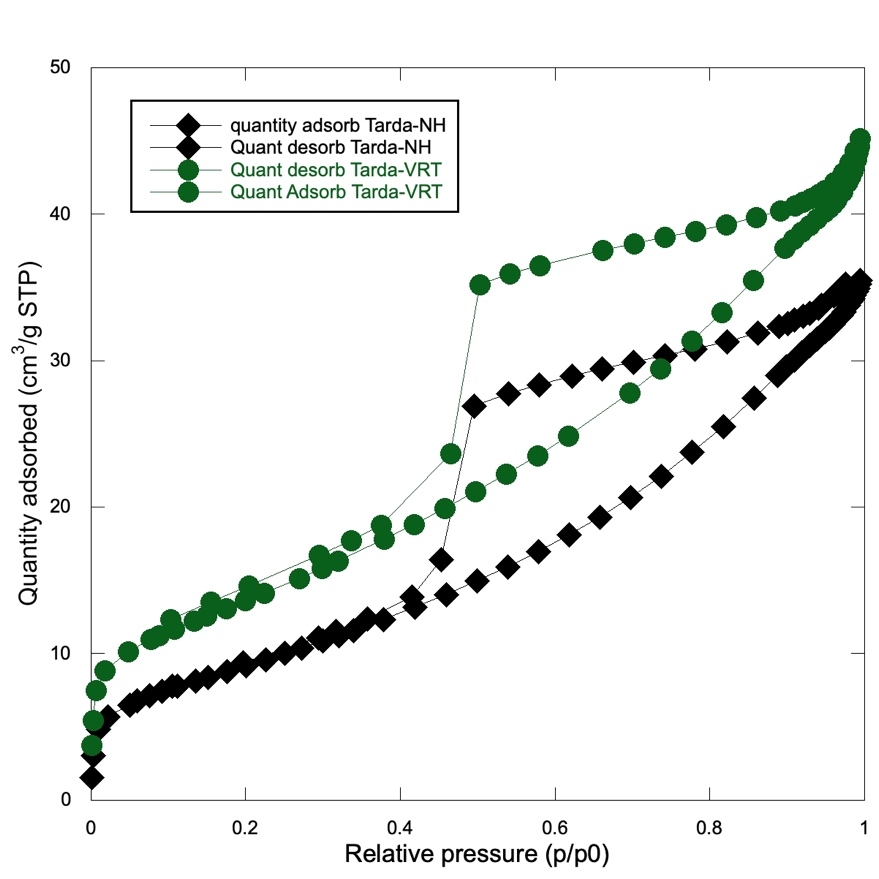


**Figure S4a.** N_2_ BET isotherm acquired at 77 K for Tarda-NH and Tarda-VRT.


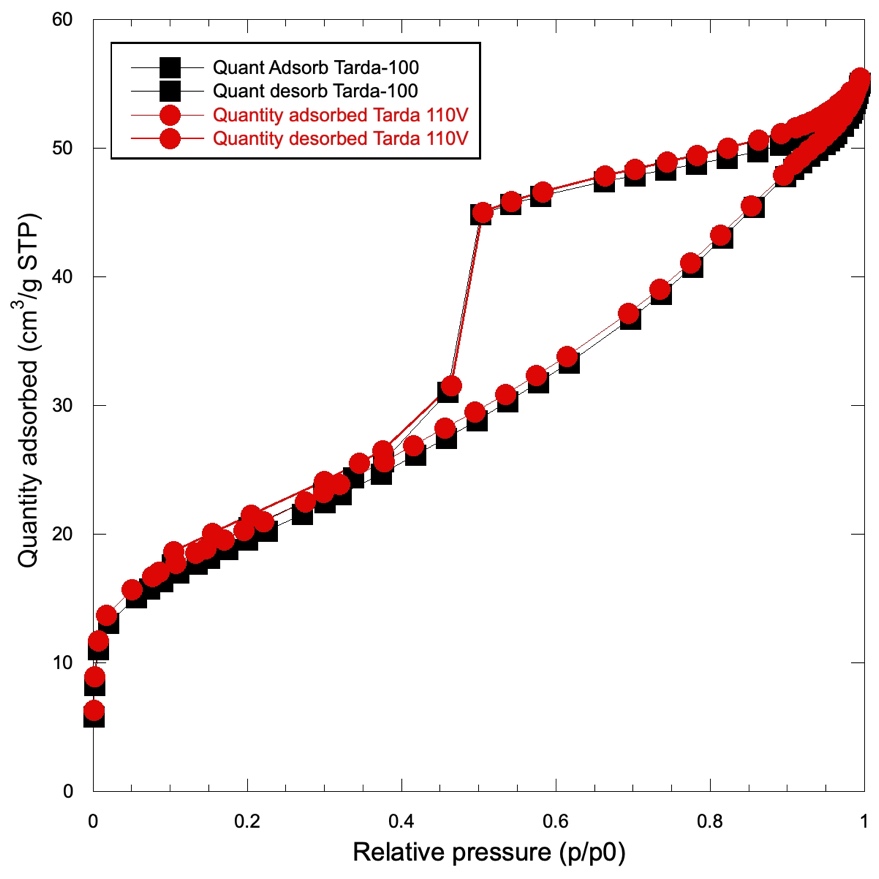


**Figure S4b.** N_2_ BET isotherms acquired at 77 K for Tarda-100 and Tarda-110V.


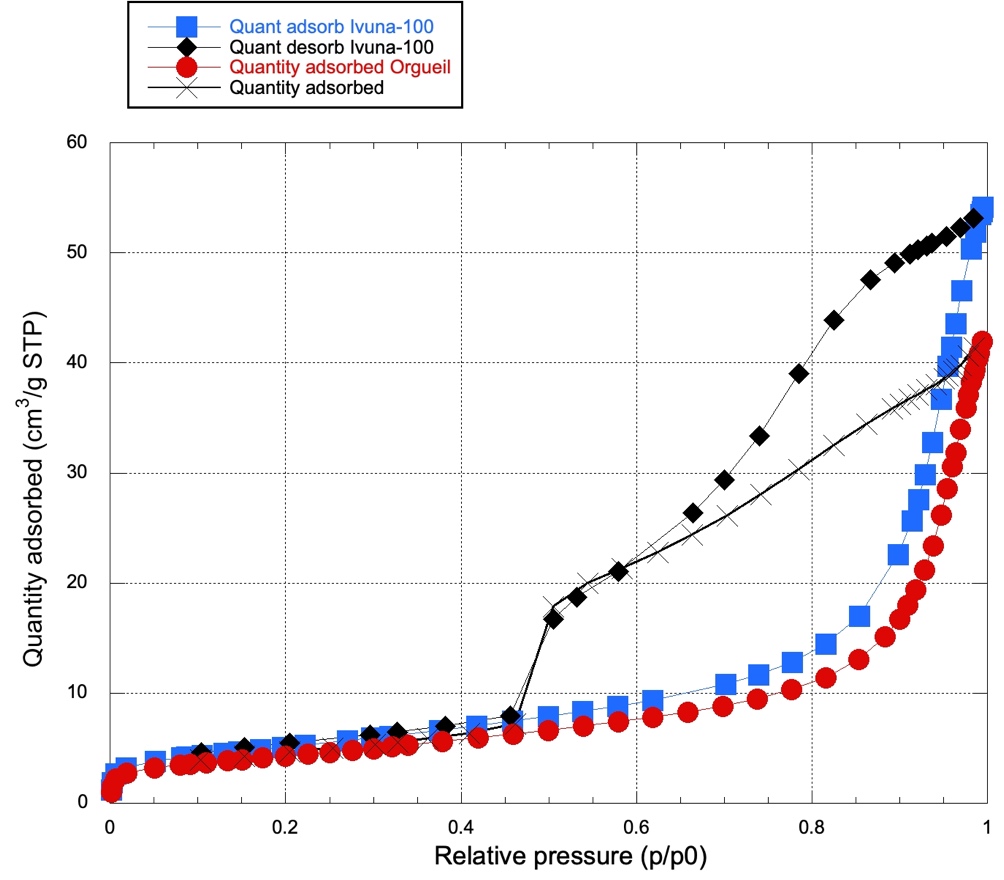


**Figure S5.** N_2_ BET isotherm acquired at 77 K for Ivuna (Adsorption isotherm – blue squares, desorption isotherm – black diamonds) and Orgueil (Adsorption isotherm – red circle, desorption isotherm – black X).


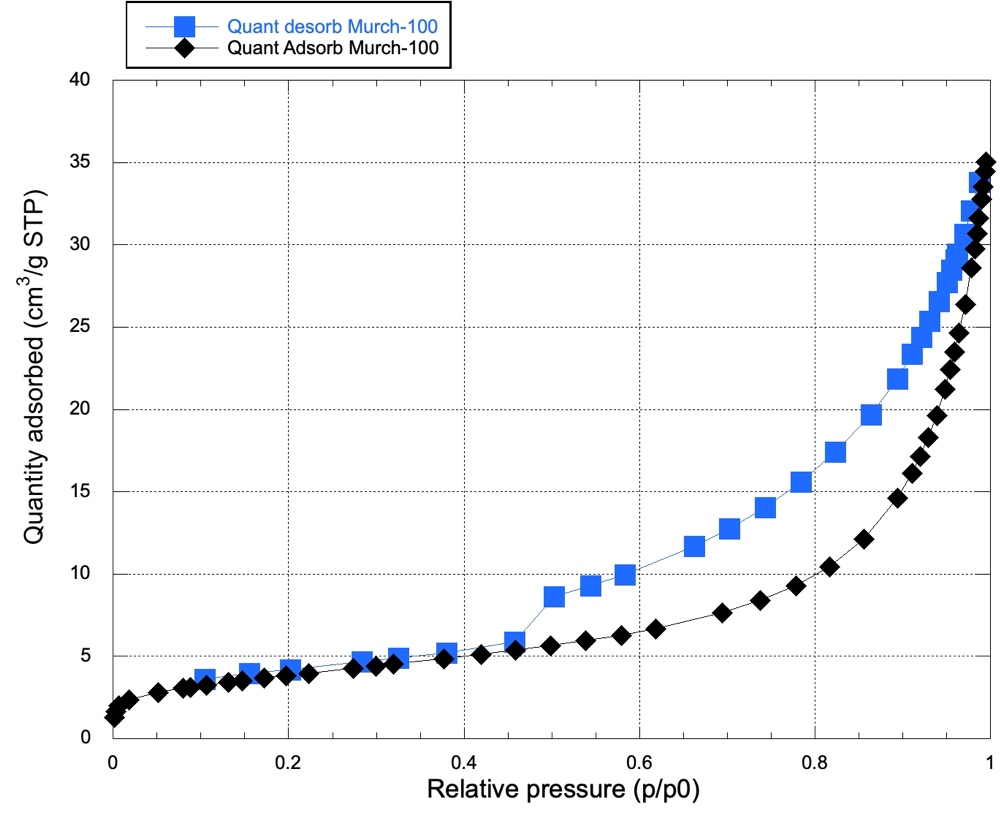


**Figure S6.** N_2_ BET isotherm acquired at 77 K for Murchison. Adsorption isotherm – black diamonds. Desorption isotherm – blue squares.

**
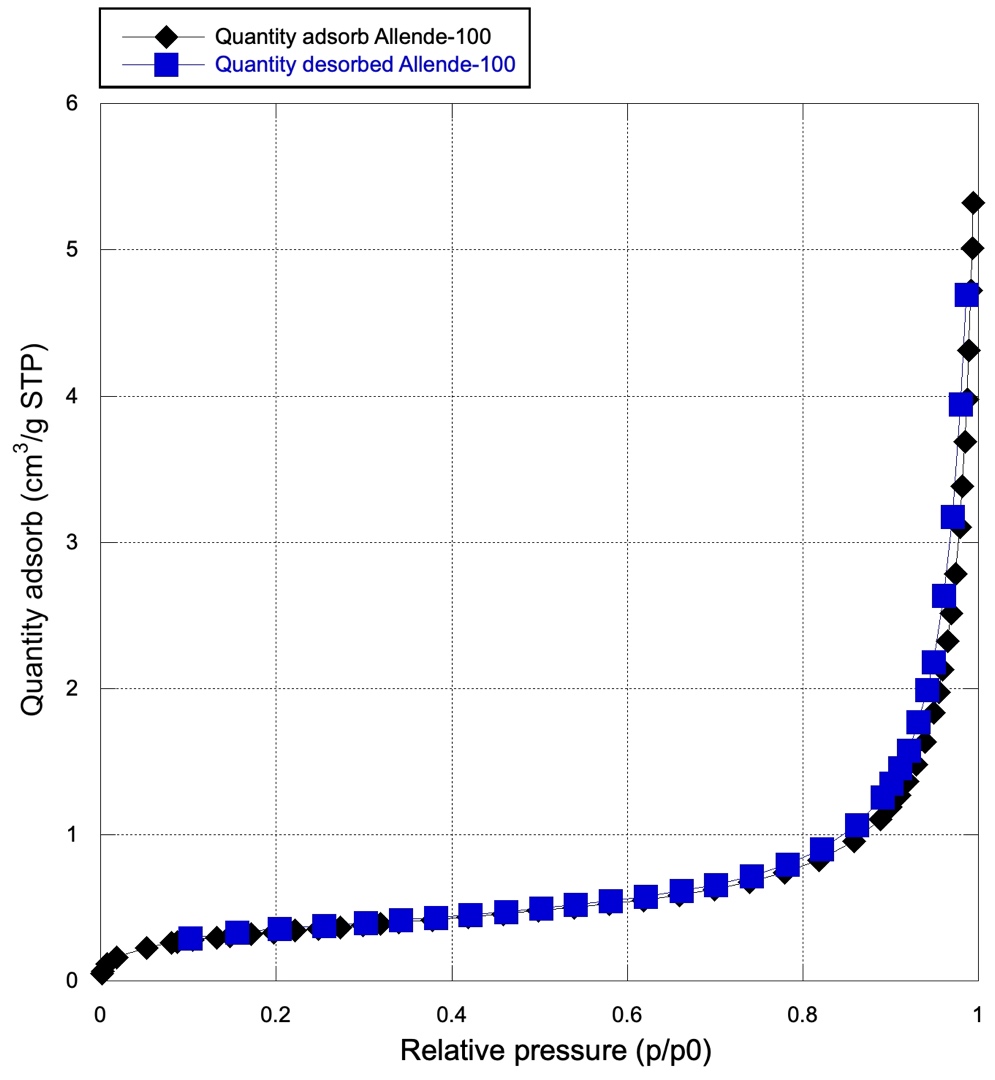
**

**Figure S7.** N_2_ BET isotherm acquired at 77 K for Allende. Adsorption isotherm – black diamonds. Desorption isotherm – blue squares.


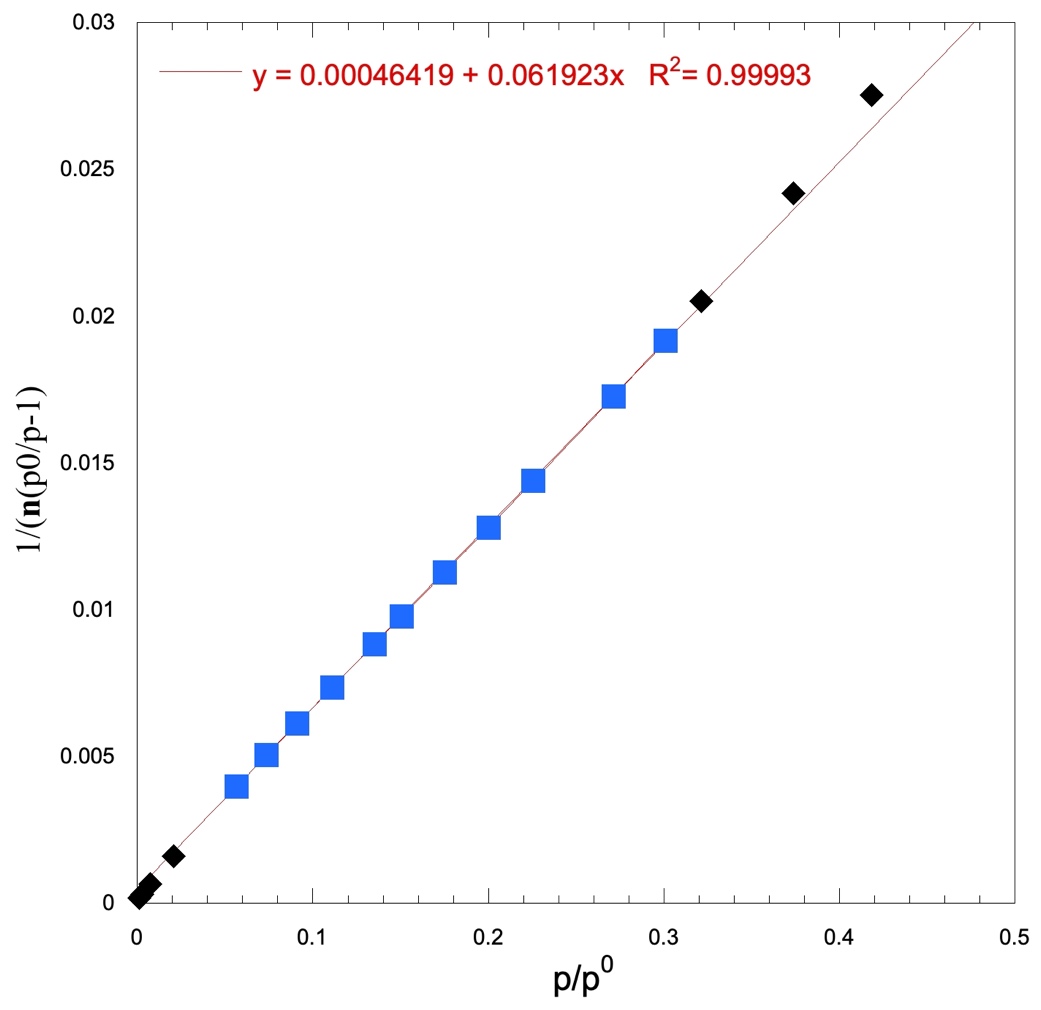


**Figure S8.** BET plot for Tarda-100 showing that the p/p^0^ values from 0.30074 to 0.056479 lie along a straight line with R^2^=0.99993 and is the region of the isotherm in which statistically the volume adsorbed corresponds to just the complete monolayer.


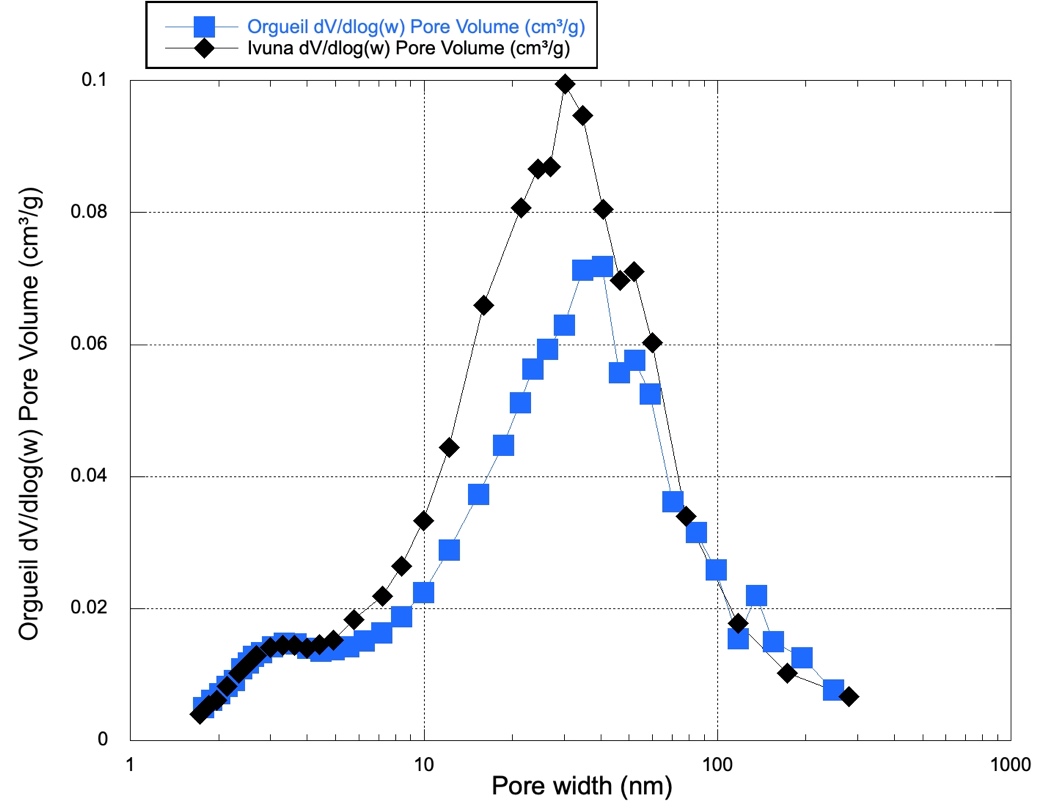


**Figure S9.** Comparison of the logarithmic differential pore-volume distribution, dV/d (log(w)) versus pore width calculated from the N_2_ BET data with the Halsey Faas correction for Orgueil (blue squares) and Ivuna (black diamonds).


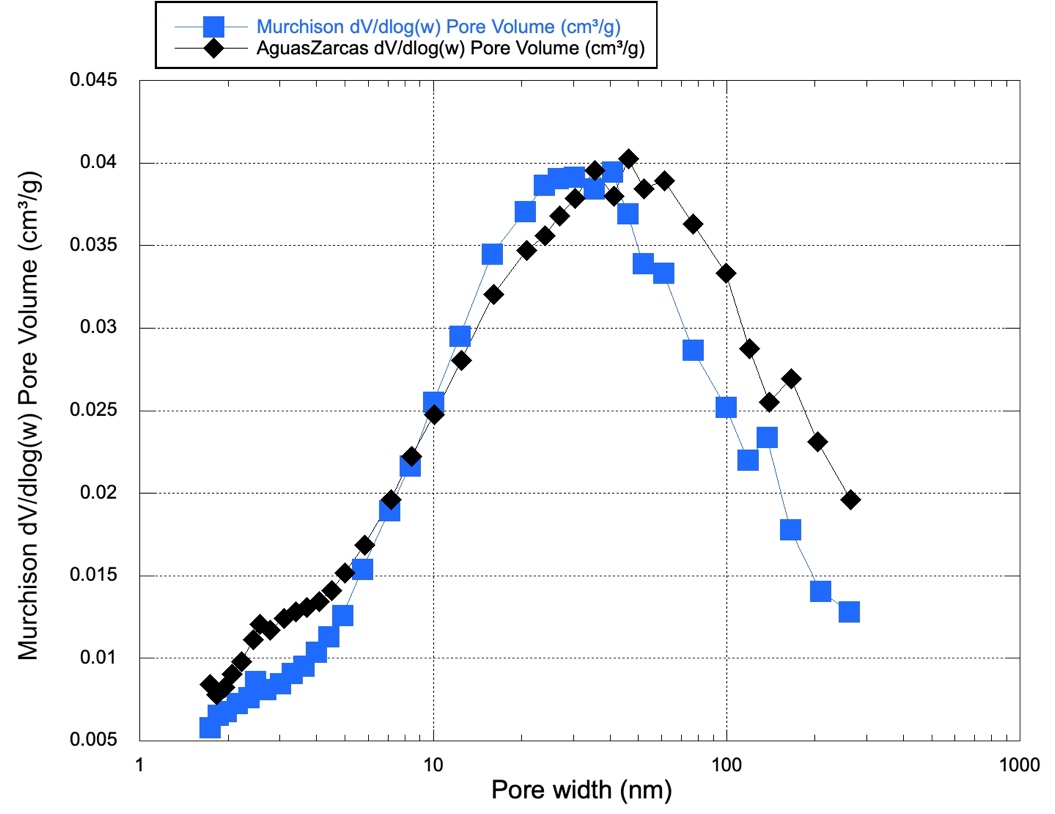


**Figure S10.** Comparison of the logarithmic differential pore-volume distribution, dV/d (log(w)) versus pore width calculated from the N_2_ BET data with the Halsey Faas correction for Murchison (blue squares) and Aguas Zarcas (black diamonds).


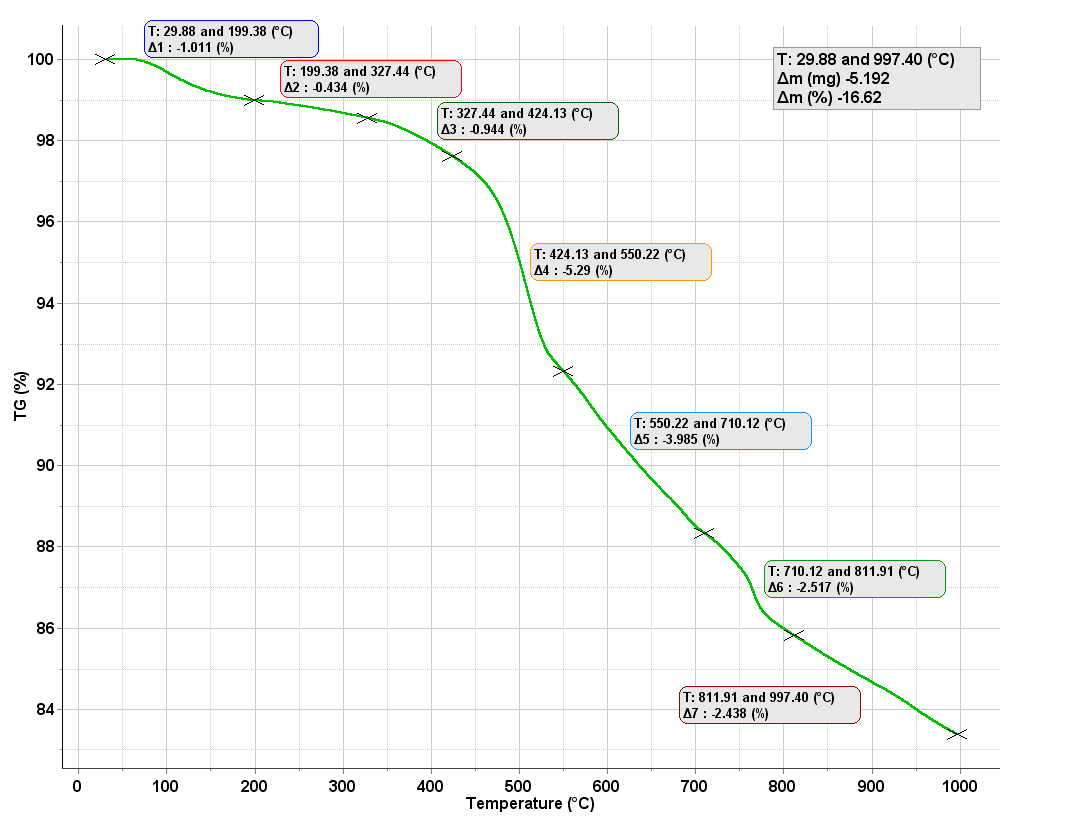


**Figure S11.** Thermogravimetric analysis (TG) for Tarda curated under N_2_ atmosphere.


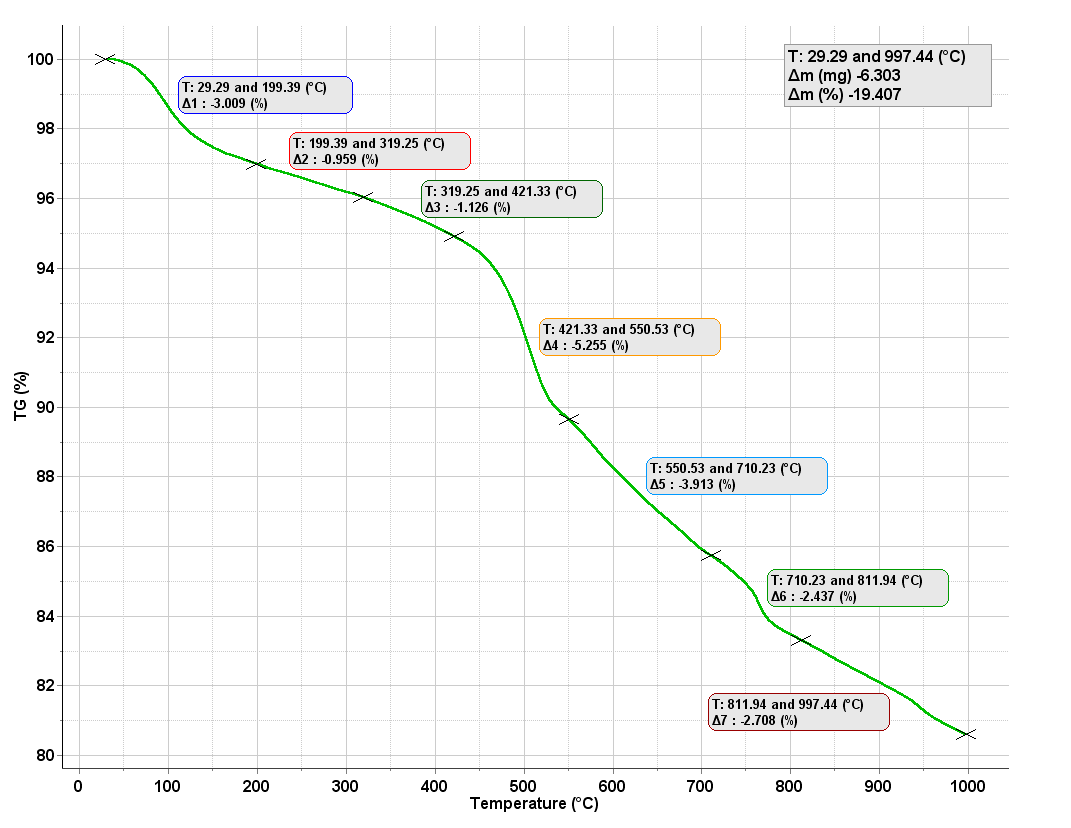


**Figure S12.** Thermogravimetric analysis (TG) for Tarda after artificial weathering in water.


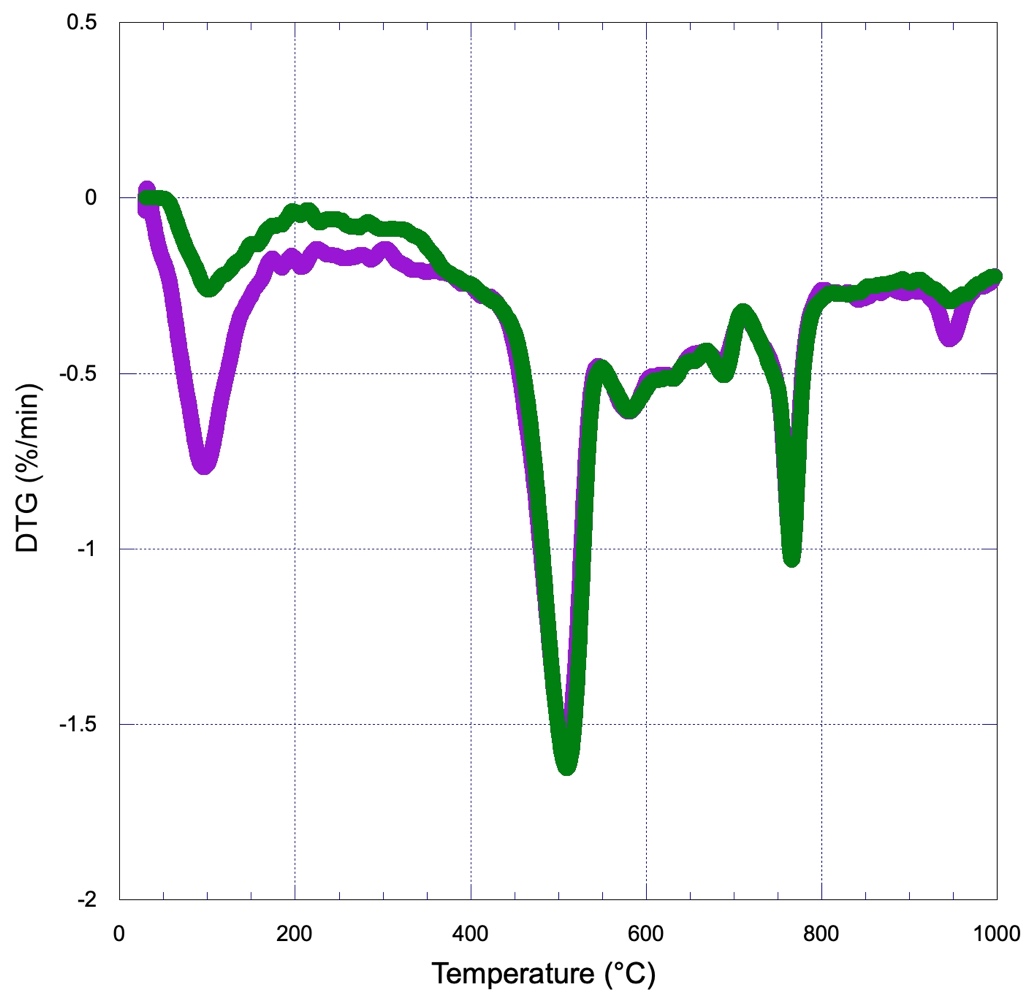


**Figure S13.** Differential thermogravimetric analysis (DTG) for Tarda curated under N_2_ atmosphere (green curve) and artificially weathered with water (purple curve).


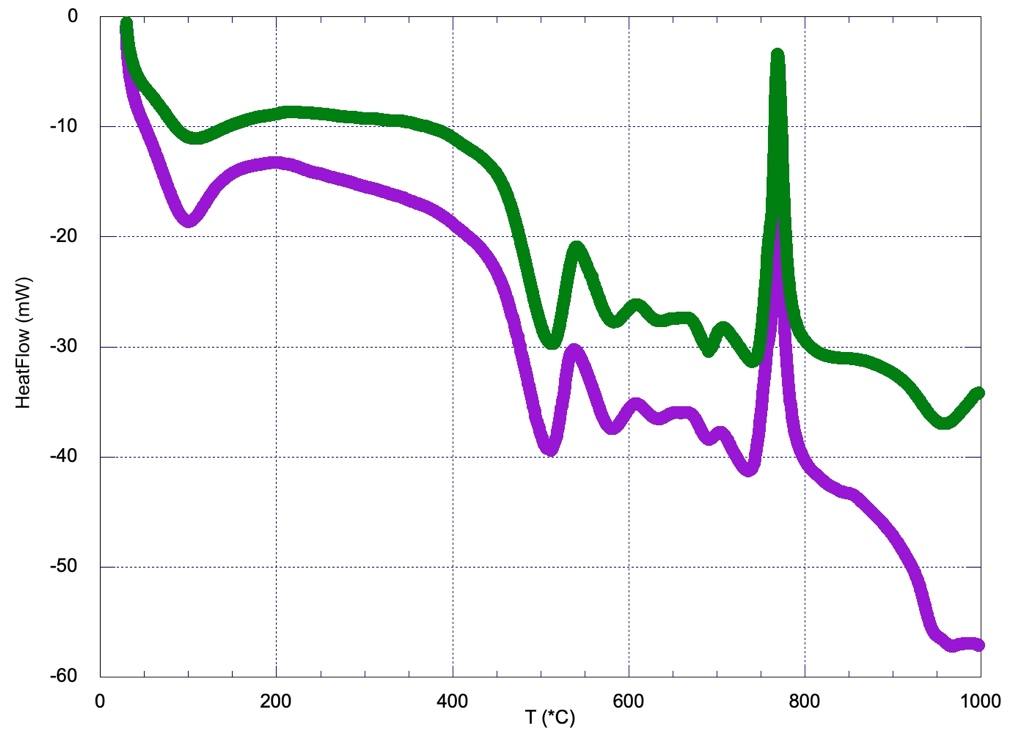


**Figure S14.** Differential scanning calorimetry (DSC) for Tarda curated under N_2_ atmosphere (green curve) and artificially weathered with water (purple curve).


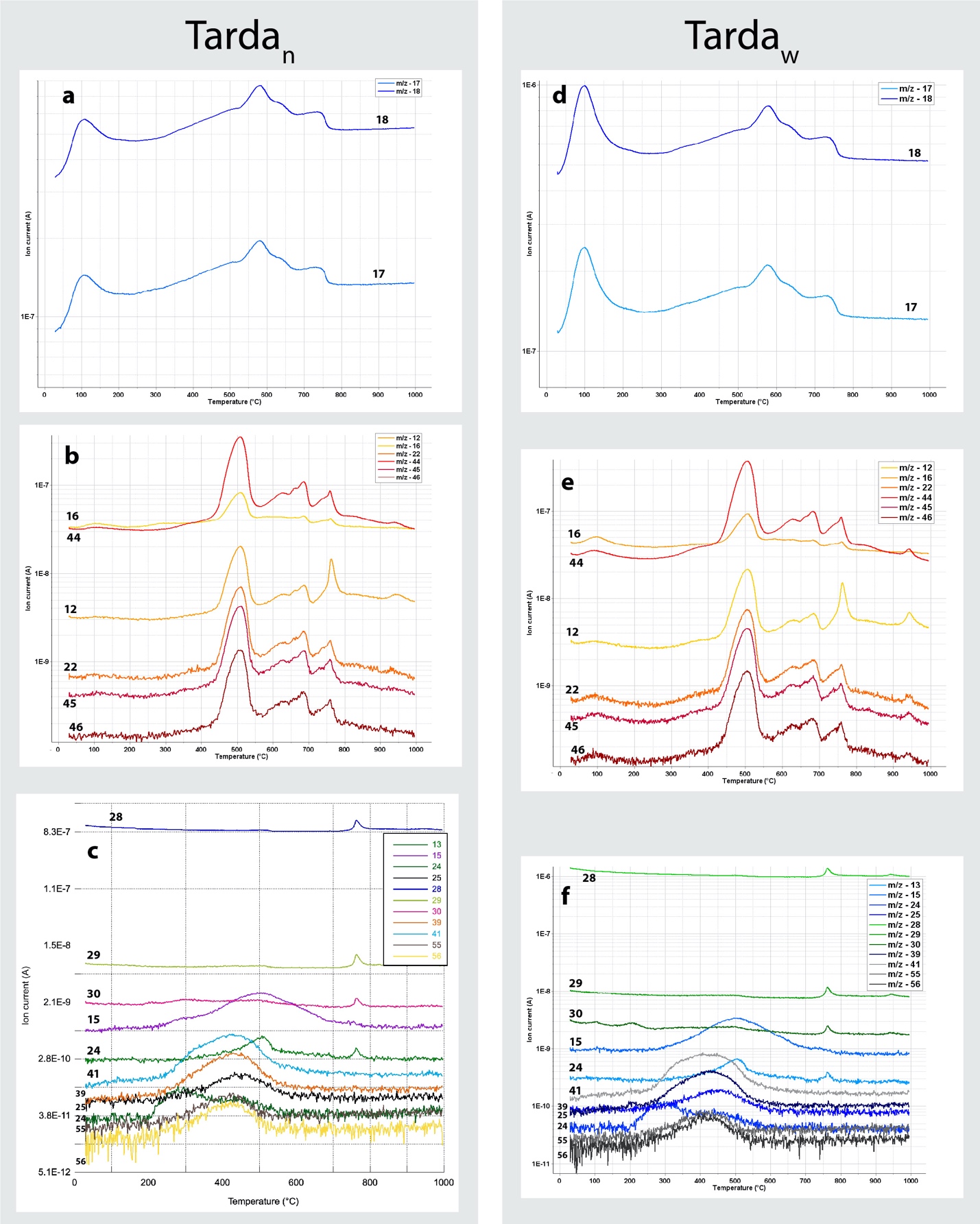


**Figure S15**. Selected MSEGA data for Tarda dry N_2_ atmosphere Tarda_N_ and Tarda mixed with distilled water and allowed to dry at room temperature – Tarda_W_. Tentative formula assignments of the detected masses are shown below.

Tentative formula assignments of the detected masses with possible sources for the ion curves are shown in Figure S15.

**Fragment (m/z) Possible formula (and source)**

***Fig. S16a,d***

17 OH^+^, NH2+ (water, amines)

18 H2O^+^ (water)

***Fig. S16b,e***

12 12C+ (organics, carbon dioxide)

16 O+ (water)

22 ^12^CO2++ (carbon dioxide)

44 ^12^CO2+ (carbon dioxide)

45 ^13^CO2+ (carbon dioxide)

46 ^12^C^18^O^16^O2+ (carbon dioxide)

***Fig. S16c,f***

13 CH^+^ (aliphatic and/or aromatic hydrocarbons)

15 CH3+ (methyl derivatives, alkyl groups)

24 C_2_^+^ (aromatic hydrocarbons)

25 C_2_H^+^ (aromatic hydrocarbons)

28 C2H4+ (aromatic hydrocarbons), CO (organic acids, esters)

29 C2H5+ (ethyl derivatives, aliphatic hydrocarbons), CHO (aldehydes)

30 CH2O^+^, NH2CH2+, C2H6+ (methyl ethers, alkyl groups)

39 C3H3+ (aromatic hydrocarbons?)

1. C3H5+ (propyl esters?)

55 C4H7+ (butyl esters?)

56 C4H8+ (polyunsaturated hydrocarbon chain)


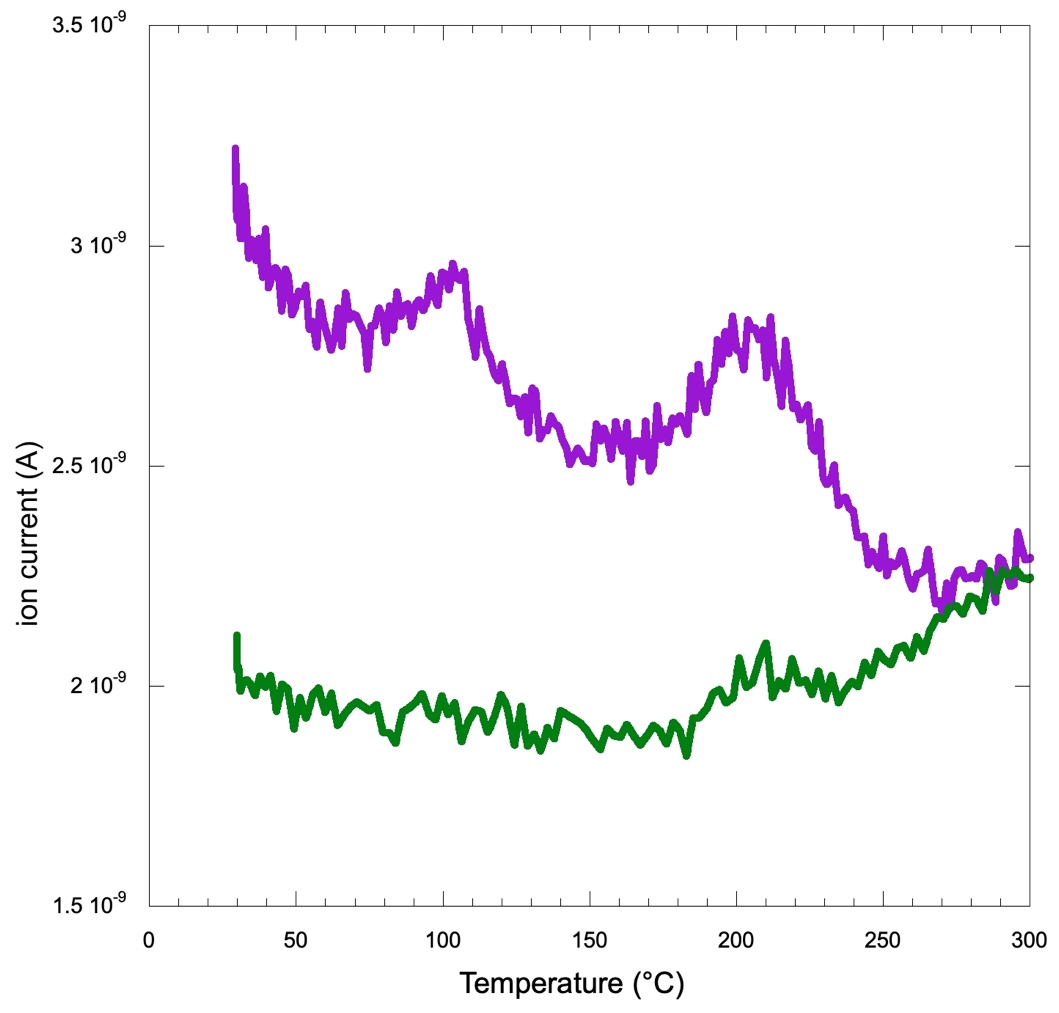


**Figure S16**. MSEGA for m/z=30 curves for Tarda curated under nitrogen (green curve) and artificially weathered with water (purple curve). Data are shown for the low-temperature region to 300 °C.


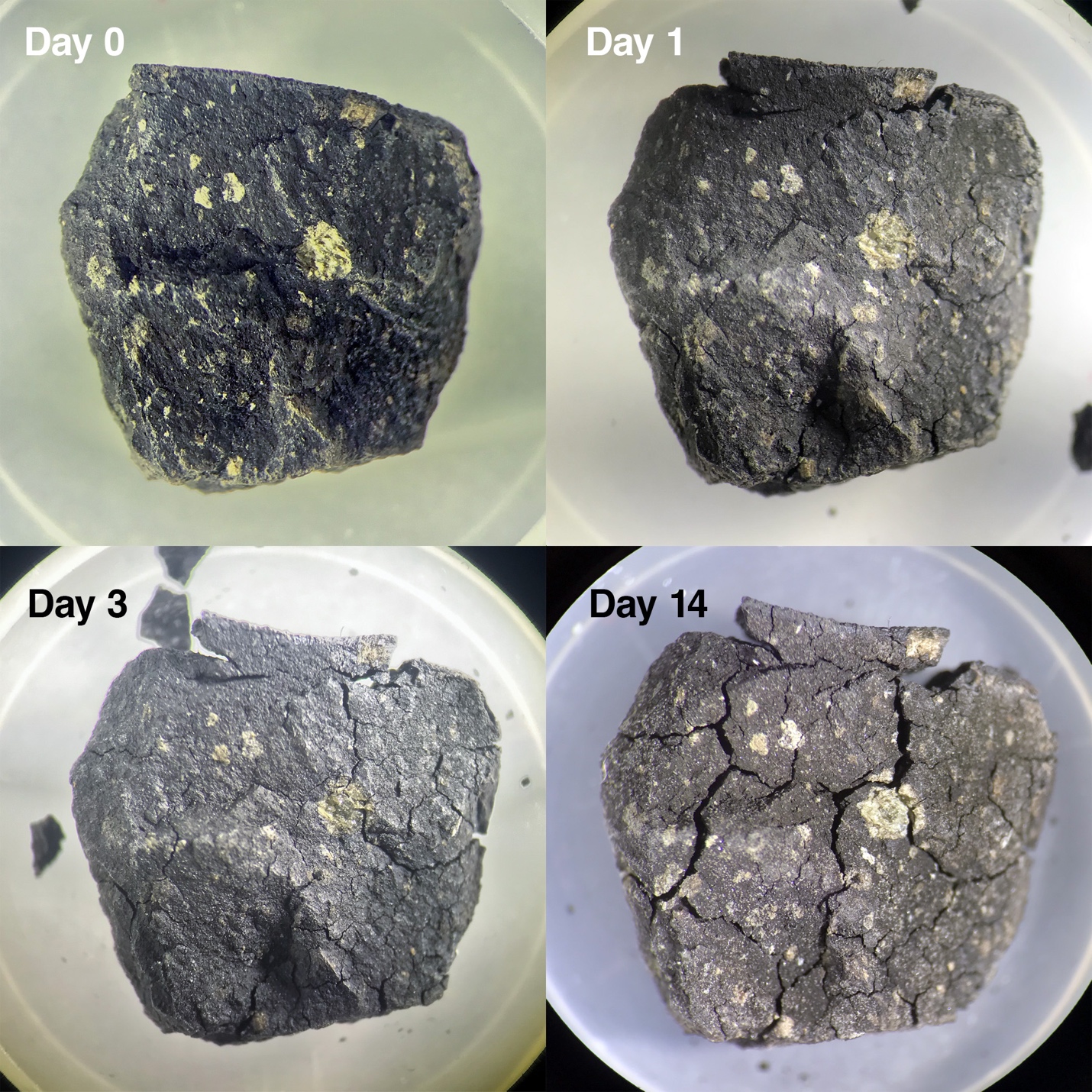


**Figure S17.** Photograph an ~0.2 g piece of Tarda directly from the N_2_ atmosphere (Day 0) and after one, three, and 14 days under 32°C and 100% RH.

**Movie S1**. The movie shows the real-time slaking and disintegration of an ~60 mg fragment of Tarda in water. Large tick bars = 1 mm. (The movie is attached as a separate file).
